# Microglial MARCO facilitates Varicella zoster virus uptake and triggers TLR2-mediated neuroinflammation

**DOI:** 10.1186/s12929-026-01256-9

**Published:** 2026-05-23

**Authors:** Ji-Soo Lim, Soo-Jin Oh, Ji-Yeun Hur, Subin Oh, Seuk-Min Ryu, Rafael T. Han, Hosun Park, Dawn M. E. Bowdish, Ok Sarah Shin

**Affiliations:** 1 BK21 Graduate Program, Department of Biomedical Sciences, Korea University College of Medicine, Seoul, Korea; 2 Department of Biological Sciences, Ajou University, Suwon, Korea; 3 Biomedical Research Division, Korea Institute of Science and Technology (KIST), Seoul, Korea; 4 Center for Advanced Biomolecular Recognition, Biomedical Research Division, Korea Institute of Science and Technology (KIST), Seoul, Korea; 5 KHU-KIST Department of Converging Science and Technology, Kyung Hee University, Seoul, Korea; 6 Department of Microbiology, College of Medicine, Yeungnam University, Daegu, Korea; 7 Department of Medicine, McMaster University, Hamilton, ON Canada; 8 Vaccine Innovation Center, Korea University College of Medicine, Seoul, Korea

**Keywords:** Varicella zoster virus, MARCO, TLR2, Microglia, Sensory neurons

## Abstract

**Background:**

Varicella zoster virus (VZV) is a human neurotropic virus that can establish latency in sensory neurons. Microglia play a complex role during neurotropic virus infections; however, their role during VZV infection remains to be determined. In the present study, we explored the role of VZV-induced alterations in the morphodynamics and function of microglia in triggering neuroinflammation.

**Methods:**

We prepared cell-free VZV and compared replication efficiencies of wild-type (YC01) and attenuated (MAV/06, MAV) VZV in two transformed human microglial cell lines (HMC3 and HIM) and human embryonic stem cell (ESC)-derived microglia (ESC-MG). Bulk RNA sequencing was used to assess molecular signatures of microglia following VZV infection in ESC-MG, and cytokine profiles were determined to further investigate neuroinflammation. To further examine the impact of VZV-induced microglial inflammation on neuronal responses, we generated ESC-derived sensory neurons (ESC-SN) and evaluated nociceptor expression and calcium flux as a readout for SN activities following microglial secretome treatment.

**Results:**

VZV upregulates its gene and protein expression and triggered morphological changes in various microglia cultures. Transcriptomic analysis of YC01-infected ESC-MG revealed a robust induction of genes associated with antiviral innate immunity, alongside a pronounced upregulation of macrophage receptor with collagenous structure (MARCO). Functional studies demonstrated that MARCO facilitates VZV uptake in microglia by binding to the viral glycoprotein E (gE) via its C-terminal scavenger receptor cysteine-rich (SRCR) domain, thereby promoting viral entry and phagocytosis. Moreover, VZV infection elicited neuroinflammation in an ORF62-dependent manner, while MARCO activation triggered toll-like receptor 2 (TLR2)-mediated inflammatory signaling. This cascade further amplified the expression of pain-associated molecular mediators in an ESC-SN model, highlighting a potential mechanistic link between microglial MARCO and VZV-triggered neuropathic processes.

**Conclusion:**

Our results show, for the first time, that microglia are susceptible to VZV infection and identify MARCO as an important mediator for regulating TLR2-mediated neuroinflammation and promoting an upregulation of factors associated with neuropathic pain.

**Graphical Abstract:**

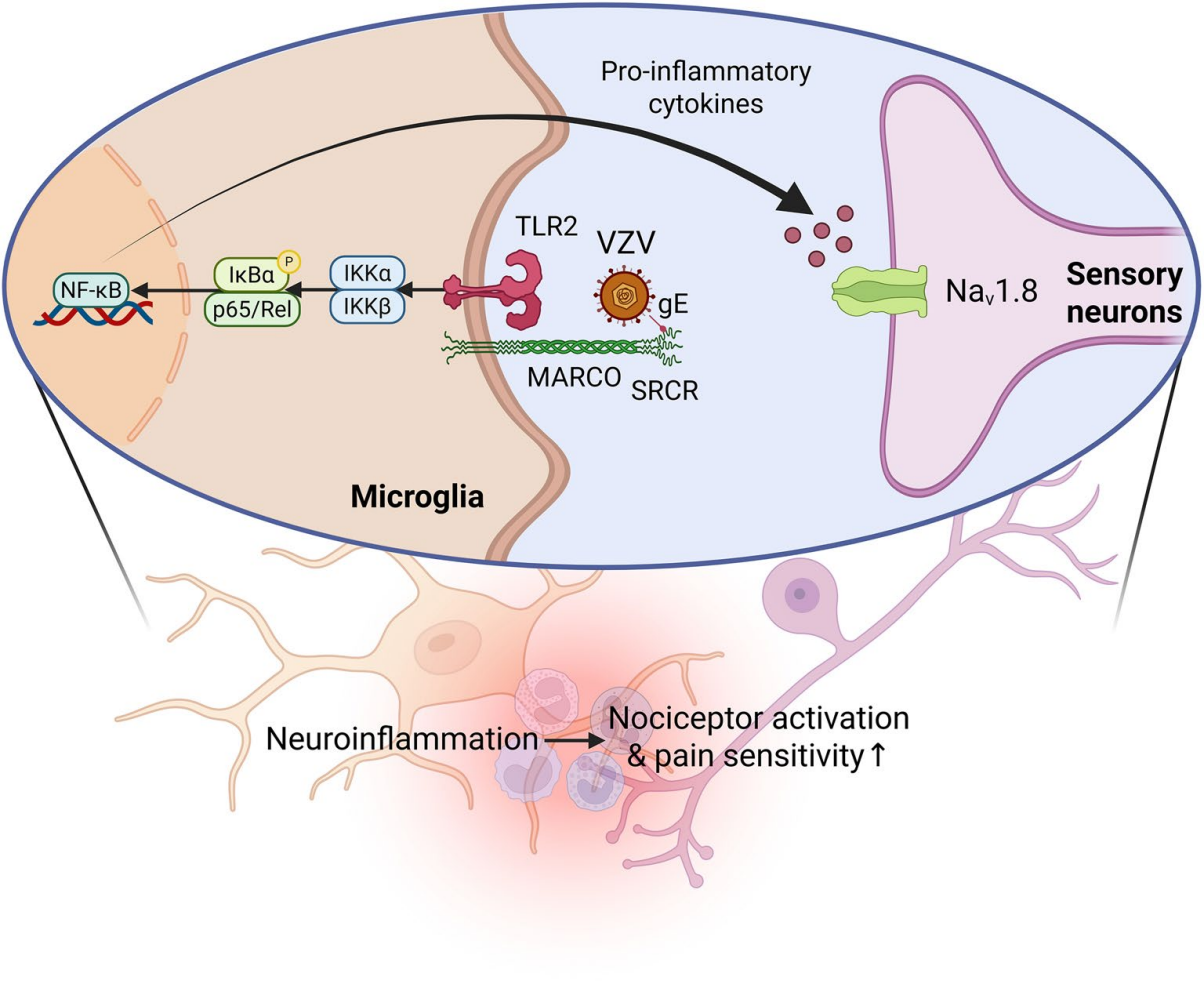

**Supplementary Information:**

The online version contains supplementary material available at 10.1186/s12929-026-01256-9.

## Introduction

Varicella zoster virus (VZV) is a neurotropic human herpesvirus that causes varicella (chickenpox) and zoster (shingles). A defining hallmark of VZV is its ability to establish lifelong latency within the host following primary infection, with latent viral genomes residing primarily in the sensory dorsal root and trigeminal ganglia [1]. VZV can lead to severe central nervous system (CNS) complications, such as meningitis, encephalitis, or acute retinal necrosis, following primary infection or reactivation of VZV [2]. The prevalence of zoster in some developed countries has decreased due to the recent introduction of highly effective recombinant vaccine, however, varicella and zoster vaccines are not still included in the national vaccination programs of many countries and that VZV-associated diseases remain prevalent. Consequently, identifying the factors that predispose individuals to CNS pathologies is critical for elucidating complex pathogenic mechanisms of VZV and developing targeted therapeutic interventions.

Administration of live-attenuated VZV vaccines has been widely used to prevent varicella, and single nucleotide polymorphisms (SNPs) that occur during the attenuation of parental strains are critical indicators of vaccine safety [3, 4]. MAV/06 (MAV) is a live-attenuated VZV vaccine strain has used in Korea that shares genetic similarities with vOka generated from pOka strain [5]. Considering that the majority of genetic variants accumulate in ORF62 region during attenuation, SNPs within this region are important indicators for determining the attenuation levels of VZV vaccines. Specifically, ORF62 from MAV strains shows considerably lower transactivation activity for ORF4, ORF28, ORF29, and ORF68 promoters than that of the wild type (WT) whereas ORF62 suppresses innate immunity by preventing IRF3 phosphorylation and interferon (IFN) production [6]. Although ORF62 interferes with IFN pathway, its potential immunomodulatory role in microglia remains unclear.

Microglia are resident immune cells in the CNS that play a pivotal role in surveillance [7]. As central innate immune defenders of the brain, microglia respond to neurotropic viruses, triggering antiviral immunity that may protect the brain from injury [8]. In particular, microglia are known to protect neuronal function through microglia-neuron crosstalk, and dysfunctional microglia with proinflammatory profiles may lead to neuronal dysfunction and damage, contributing to the pathogenesis of various neurological diseases [8, 9]. Recently, we and others have delineated the association between herpesvirus infection and neurodegenerative phenotypes via microglial inflammation [10–12], providing effective strategies to manage viral encephalitis and prevent long-term neurological complications and neurodegenerative diseases. While several studies have characterized the molecular impact of VZV infection on neurons and neuronal stem cells [13–17], the role of microglia during VZV infection remains to be determined.

Given that VZV lacks a suitable animal model, in vitro systems are essential for dissecting its cellular mechanisms. We used various microglial cell lines and embryonic stem cell (ESC)-derived human microglia (ESC-MG) to comprehensively analyze microglial gene signatures, phenotypes, and functions in response to cell-free VZV infection. Here, we report that YC01, but not MAV, induces robust changes in microglial morphology, phenotype, and function. Furthermore, microglial macrophage receptor with collagenous structure (MARCO)-toll-like receptor 2 (TLR2) axis promotes neuroinflammation and contributes to increased neuronal excitability in an ESC-derived sensory neuron (ESC-SN) model. Our findings provide the first evidence at how microglia are potential mediators of VZV-induced neurological manifestations and, consequently, can be targets for therapeutic strategies against neurological diseases with VZV.

## Materials and methods

### Cell culture and reagents

HMC3 (CRL-3304), HEK293T (CRL-3216), SH-SY5Y (CRL-2266), MeWo (HTB-65), and THP1 (TIB-202) cells were obtained from American Type Culture Collection. SV40 cells (human immortalized microglia; HIM) were obtained from Applied Biological Materials Inc.. HMC3, HEK293T, and SH-SY5Y were cultured in Dulbecco’s modified Eagle medium (DMEM; CM002-050, GenDepot) supplemented with 10% fetal bovine serum (FBS; F0900-050, GenDepot) and 1% penicillin/streptomycin (P/S; 15140-122, Gibco). MeWo cells were maintained in minimum essential medium (MEM; MCL-052, Serana) supplemented with 10% FBS and 1% P/S. HIM cells were cultured in Prigrow III medium (TM003, Applied Biological Materials Inc.) supplemented with 10% FBS and 1% P/S. THP1 cells were cultured in RPMI 1640 medium (A10491-01, Gibco) supplemented with 10% FBS, 1% P/S, and 0.055 mM 2-mercaptoethanol (21985-023, Gibco), and differentiated into macrophages in the presence of 50 ng/mL phorbol 12-myristate 13-acetate (PMA; P1585, Sigma-Aldrich). A stable HEK293T cell line expressing VZV glycoprotein E (gE) was generated in a previous study, and stable expression of VZV gE was confirmed using immunoblotting and flow cytometry prior to use [18]. MARCO-knockout (KO) HMC3 cells were generated using CRISPR-Cas9 gene editing. After CRISPR-Cas9 transfection, single cell clonal expansion was performed to establish individual clones. Genomic DNA from each clone was used to construct sequencing libraries, and the knockout efficiency was assessed by sequencing, which confirmed complete deletion at the target locus.

MARCO-blocking peptide was purchased from Biosynth (33R-7477). Lipopolysaccharide (LPS) from *E. coli* O111:B4 (L2630), polycytidylic acid potassium salt (poly(C)), polyinosinic acid potassium salt (poly(I), P4154), and capsaicin (211275) were purchased from Sigma-Aldrich. Human TNF-α recombinant protein was purchased from PeproTech (300-01A) and staurosporine (1258) was purchased from Tocris.

### Generation of human embryonic stem cells (ESC)-derived microglia (ESC-MG)

Embryoid bodies (EBs) were generated from H1 human ESCs to obtain microglia progenitors and mature microglia as described previously, with minor modifications [12]. Briefly, ESCs were seeded in ultra-low attachment 96-well plates (7007, Corning) with mTeSR1 medium (85850, STEMCELL) supplemented with 50 ng/mL BMP4 (795606, PeproTech), 50 ng/mL VEGF (583704, BioLegend), 20 ng/mL SCF (573902, BioLegend), and 10 µM ROCK inhibitor (1254, Tocris). On day 4, EBs with a diameter of 700–800 µm were transferred to ultra-low attachment 6-well plate containing X-VIVO 15 media (02-060F, Lonza) supplemented with 2 mM GlutaMAX (35050-061, Gibco), 100 U/mL Antibiotics-Antimycotic (CA002-010, GenDepot), 0.055 mM 2-mercaptoethanol (21985-023, Gibco), 50 ng/mL SCF, 50 ng/mL M-CSF (574804, BioLegend), 50 ng/mL IL3 (578004, BioLegend), 50 ng/mL FLT3 (GMP-10315-HNAE1-L-AF, Sino Biological), and 5 ng/mL TPO (763702, BioLegend). On day 11, the medium was replaced with the X-VIVO 15 medium supplemented with 2 mM GlutaMAX, 100 U/mL antibiotic–antimycotic, 0.055 mM 2-mercaptoethanol, 50 ng/mL FLT3, 50 ng/mL M-CSF, and 25 ng/mL GM-CSF. When microglial progenitors were visible on days 18 and 25, the cells were collected and maintained in RPMI1640/GlutaMAX medium (61870036, Gibco) supplemented with 100 ng/mL IL34 (200–34, PeproTech) and 10 ng/mL M-CSF for microglial maturation. The expression of CD14 (325603, BioLegend) and CX3CR1 (341611, BioLegend) during differentiation was assessed by flow cytometry for quality control.

### Generation of human ESC-derived sensory neuron (ESC-SN)

Sensory neurons (SNs) enriched for nociceptors were differentiated from H1 human ESC according to a previously published protocol with minor modifications [19]. Briefly, undifferentiated ESCs were maintained in Matrigel (354277, Corning)-coated dishes in Essential 8 medium (A1517001, Gibco) until they reached 80–90% confluency. Neural crest cell (NCC) differentiation was initiated by treating the cells with Essential 6 medium (A1516401, Gibco) supplemented with 10 μM ROCK inhibitor (1254, Tocris), 300 nM CHIR99021 (4423, Tocris), 10 μM SB431542 (1614, Tocris), and 1 ng/mL BMP4 (795606, PeproTech). From day 2, the medium was replaced with NCC differentiation media containing 0.75 μM CHIR99021, 10 μM SB431542, 2.5 μM DAPT (2634, R&D Systems), and 2.5 μM SU5402 (SML0443, Sigma-Aldrich). NCCs were harvested using Accutase (A1110501, Gibco), treated with DNase I (10104159001, Roche), and replated onto poly-L-ornithine (P3655, Sigma-Aldrich)/laminin-1 (23017015, Gibco)/fibronectin (356008, Corning)-coated 24-well plates at a density of 2.5 × 10^5^ cells/cm^2^ on day 12, which yielded a mixed SN population with nociceptors, mechanoreceptors, and proprioceptors. Cells were then cultured in neurobasal medium (21103049, Gibco) supplemented with N2 (17502048, Gibco), B27 (17504-044, Gibco), 2 mM L-glutamine (25030-081, Gibco), 25 ng/mL NGF (450-01, Gibco), 20 ng/mL BDNF (11166-BD, R&D Systems), 20 ng/mL GDNF (450-10, Gibco), 0.125 μM retinoic acid (R2625, Sigma-Aldrich), antibiotic–antimycotic (CA002-010, GenDepot), and 1 μM DAPT. The medium was refreshed every 2–3 days. By day 30, neurons exhibited a dorsal root ganglion (DRG)-like profile with nociceptors being the predominant subtype. SOX10 (sc-365692, Santa Cruz) and BRN3A (MAB1585, Merck Millipore) expression during differentiation was assessed by immunofluorescence and RT-qPCR for quality control.

### Cell-free VZV preparation and titration

VZV strain YC01 was isolated from a zoster patient and propagated as previously described [18, 20], whereas cell-free MAV/06 (MAV) was obtained from GC Biopharma (Yongin, Korea). Cell-free VZV was prepared using modified methods suggested by Sloutskin et al. [21]. Briefly, cell-associated VZV was inoculated into MeWo cell monolayers. Infected MeWo cells were harvested at 70–80% cytopathic effects (CPE) and scraped using PBS-sucrose-glutamate-serum buffer (PSGC) adapted from Harper et al. [22]. The resultant solution was obtained by repeated freezing and thawing three times to disrupt the cellular components. Sonication was performed using a Q800R3 sonicator (QSONICA) to obtain the viral particles and discard as much cellular debris as possible. After centrifugation at 3500 rpm for 15 min at 4 °C, the supernatant containing cell-free VZV was collected and stored at − 80 °C. For titration of cell-free VZV, viral diluents were inoculated into MeWo cells for 1.5 h and overlaid with MEM containing 1% carboxymethylcellulose (C4888, Sigma-Aldrich). Cells were stained with 0.5% crystal violet solution in 20% methanol after 6–7 days, and cell-free VZV titer was calculated using a plaque assay.

### Cell viability assay

Cytotoxicity in VZV-infected cells was measured using CCK-8 assay (CK04-11, Dojindo) according to the manufacturer’s protocol, and absorbance was measured at 450 nm using a Varioskan LUX Multimode Microplate Reader (Thermo Fisher).

### Bulk RNA-seq and data analysis

Total RNA was isolated using TRIzol reagent (15596018, Invitrogen). RNA samples with RNA integrity number > 7.0 were sequenced as paired-end 150 × 150 bp on a NovaSeq^®^ 6000 System (Illumina, Inc.).The quality of the raw fastq files was assessed using FastQ [23] and the trimmed reads were mapped to the reference genome using STAR [24]. Read quantification was performed using Salmon [25], and the gene abundance of mapped reads was calculated using human gene annotation from Ensembl with Feature Counts v1.6.0. Differential expression analysis and graphical visualization were performed using the ExDEGA software (Ebiogen Inc., Korea). Differentially expressed genes (DEGs) were subjected to Gene Ontology (GO) enrichment and Kyoto Encyclopedia of Genes and Genomes (KEGG) pathway analyses using DAVID (v6.8; https://david.ncifcrf.gov/). The datasets generated during the current study can be accessed by GEO accession number GSE309260.

### Plasmids, siRNA, and transfection

pSG5-HA-ORF62-wild-type pOka and pSG5-HA-ORF62-mutant (including M99T, S628G, R958G, V1197A, I1260V, L1275S mutations) were kindly provided by Prof. Jin Hyun Ahn (Sungkyunkwan University, Korea) [6]. Full-length MARCO-Myc and scavenger receptor cysteine-rich (SRCR) domain-lacking MARCO-Myc plasmids were previously reported [26]. pTargeT-VZV gE (a gift from Charles Grose; Addgene plasmid #60845) has been previously described [27], while pCMV3-hTLR2-HA plasmid was purchased from Sino Biological (HG10061-CY). HEK293T cells were transiently transfected with DNA plasmids using polyethyleneimine (23966, Polysciences), whereas HMC3 cells were transiently transfected with DNA plasmids using Lipofectamine 2000 (11668019, Invitrogen), according to the manufacturer’s instructions. For RNA interference, the cells were seeded in 6-well plates and cultured until 70% confluence was reached on the day of transfection. Transient transfections with MARCO-specific siRNAs (Bioneer, Korea) were performed using the RNAiMAX transfection reagent (13778075, Invitrogen) according to the manufacturer’s protocol.

### Luciferase reporter assay

The luciferase reporter assay was performed as previously described [28]. Briefly, HEK293T or HMC3 cells were seeded into 96-well plates and transiently transfected with 100 ng of NF-κB or IP-10 luciferase reporter plasmids in combination with different plasmids. Following incubation, luciferase activity was quantified using Steady-Glo Luciferase Assay System (E2510, Promega) in accordance with the manufacturer’s instructions.

### Immunofluorescence imaging

Cells were fixed with 4% paraformaldehyde, permeabilized with 0.1% Triton X-100 diluted in PBS, and blocked with 2.5% bovine serum albumin (BSA; A8412, Sigma) for 30 min at each step. Primary antibodies against HA-Tag (3724, Cell Signaling), NF-κB p65 (SC-8008, Santa Cruz), MARCO (SC-398053, Santa Cruz), VZV gE (SC-56995, Santa Cruz), Myc-Tag (10828-1-AP, Proteintech), SOX10 (sc-365692, Santa Cruz), BRN3A (MAB1585, Merck Millipore), or SCN10A (55334-1-AP, Proteintech) were prepared in 0.5% BSA in PBS and incubated with cells for 1 h, followed by secondary antibody incubation using anti-mouse Alexa594 (A11005, Invitrogen), and anti-rabbit Alexa488 (A11008, Invitrogen). For nuclear staining, cells were incubated with 1 µg/mL 4ʹ,6-diamidino-2-phenylindole (DAPI) for 10 min. The stained cells were imaged using a confocal microscope (LSM900, Carl Zeiss) and analyzed using a Zen desk (Carl Zeiss). For the detection of reactive oxygen species (ROS) and cell death, cells were stained with 10 μM 2′,7′-dichlorofluorescein diacetate (DCF-DA; D399, Invitrogen) and with 5 μg/mL propidium iodide (PI; P4170, Sigma-Aldrich) in serum-free media for 30 min. Fluorescence images were acquired using an EVOS FL imaging system and merged with the DIC images for analysis. For quantification of degenerating neurons, cells were stained with Fluoro-Jade C (FJC; TR-100-FJT, Biosensis), and FJC-positive cells were counted from at least 200 cells per condition, compiled from at least three experiments and expressed as the percentage of % FJC-positive cells relative to total DAPI-positive cells.

### Microglial morphometric analysis

Cells were fixed with 4% paraformaldehyde, permeabilized with 0.1% Triton X-100 diluted in PBS, and blocked with 2.5% BSA for 20 min at each step. Primary antibodies against Iba1 (019-19741, FUJIFILM Wako) were prepared in 0.5% BSA in PBS and incubated with cells for 1 h, followed by secondary antibody incubation with anti-rabbit Alexa 488 (A11008, Invitrogen). Stained cells were imaged using a confocal microscope and analyzed using Zen desk (Carl Zeiss) and Imaris 10.1.0 3D analysis software (Oxford Instruments). Morphometric analysis of surface area and filament length was performed on Iba1-positive microglia whose processes were entirely within the 3D Z-stack.

### Flow cytometry

Fluoresbrite BB Carboxylate Microspheres (6.00 µm) (19102, Polysciences) were used as previously described to evaluate microglia activation and phagocytic activity towards latex beads [12]. Pre-opsonized beads were added to the cells and incubated for 1.5 h, and cells were washed three times to remove any remaining beads from the surface of the microglia. Phagocytosis of the latex beads was quantified using flow cytometry (BD LSR Fortessa X-20 flow cytometer). To measure surface protein expression of microglia, cells were harvested and stained using anti-CD45 (563880, BD Biosciences), anti-CD68 (333809, Biolegend), anti-CD14 (325603, BioLegend), anti-CD36 (336203, BioLegend), anti-CX3CR1 (341,611, BioLegend), anti-TREM2 (MAB-17291, R&D Systems), anti-AXL (8661, Cell Signaling), MARCO Monoclonal Antibody (PLK-1) (12-5447-42, eBioscience) and anti-TLR2 (14-9922-82, Invitrogen) followed by secondary antibody incubation using anti-rat Alexa 488 (A11006, Invitrogen), anti-mouse Alexa 488 (A11001, Invitrogen), and anti-rabbit Alexa 488 (A11008, Invitrogen).

### Immunoblot analysis

Cells were lysed in ice-cold immunoprecipitation assay (RIPA) buffer (R0278, Sigma-Aldrich) containing protease and phosphatase inhibitors (11836170,001 and 04906845001, Roche). Lysates were centrifuged at 13,000 rpm to remove cellular debris and quantified using a BCA assay (23225, Thermo Fisher). The samples were subjected to 8–15% SDS-PAGE and transferred onto polyvinylidene difluoride (PVDF) membranes (IPVH00010, Merck). After blocking with 5% skim milk in Tris-buffered saline (TBS) supplemented with 0.1% Tween-20 (TBS/Tw) for 1 h, membranes were incubated with primary antibodies at 4 ºC overnight. The following antibodies were used: VZV gE (SC-56995; 1:1000, Santa Cruz), HA (3724; 1:2000, Cell Signaling), Actin (AM1021B; 1:2000, Abcepta), MARCO (SC-398053; 1:1000, Santa Cruz), Myc-Tag (10828-1-AP; 1:2000, Proteintech), and NF-κB Pathway Antibody Sampler Kit (9936; 1:2000, Cell Signaling). Primary antibodies were conjugated with secondary horseradish peroxidase (HRP)-conjugated anti-mouse or rabbit antibodies, followed by imaging using Fusion Solo Imaging System (Vilber Lourmat STE) and quantification using ImageJ software (National Institutes of Health).

### Co-immunoprecipitation (Co-IP)

HEK293T, seeded in 60-mm dishes, were transfected with the indicated plasmids, incubated for 24 h, and lysed in immunoprecipitation lysis buffer (87787, Thermo Fisher) supplemented with a complete protease inhibitor cocktail (11836170,001, Roche). Dynabeads Protein G (10003D, Thermo Fisher) was incubated with 1 μg primary antibodies against Myc (10828-1-AP, Proteintech) for 1 h for conjugation. After removing cell debris via centrifugation, the antibody-conjugated beads were incubated with 500 μg of cell lysates overnight at 4 °C, with rotation. After washing, immunoprecipitants were eluted in 2 × Laemmli sample buffer or SDS sample buffer and analyzed using immunoblotting to detect interactions between proteins.

### Quantitative RT-PCR

Total RNA was extracted using TRIzol reagent (15596018, Invitrogen). cDNA was synthesized from 500 to 2000 ng of RNA using a High-Capacity cDNA Reverse Transcription Kit (4368814, Applied Biosystems). Quantitative PCR was performed with the Power SYBR Green Master Mix (4367659, Applied Biosystems) in a QuantStudio 1 Real-time PCR system instrument (Thermo Fisher) under the following conditions: 95 °C for 10 min, followed by 40 cycles at 95 °C for 30 s and 60 °C for 1 min. The primer sequences are provided in Table S1 [18, 29]. Host gene expression was quantified by 2^−ΔΔCt^ method, which levels normalized to the endogenous control *GAPDH*, whereas 2^−ΔCt^ method was adopted to analyze viral gene expression.

### Enzyme-linked immunosorbent assay (ELISA)-based binding assay

Binding of recombinant human MARCO to VZV glycoproteins was assessed using an ELISA-based assay. Briefly, recombinant VZV gE protein (40907-VNAS-E-100, Sino Biological), recombinant VZV gB protein (40958-VNAS-100, Sino Biological) or bovine serum albumin (BSA) were coated onto 96-well high-binding polystyrene plates (2592, Corning) at serial dilutions ranging from 0 to 5 μg/mL in PBS (pH 7.4) and incubated overnight at 4 °C. Plates were then washed with PBS containing 0.05% Tween-20 (PBST) and blocked with 1% BSA in PBS for 1 h at room temperature. Recombinant human MARCO protein with an N-terminal His tag (7586-MA-050, R&D Systems) was added at a final concentration of 2 μg/mL and incubated to allow binding to plate-bound proteins. After washing three times with PBST, bound MARCO was detected using a mouse monoclonal anti-His tag antibody (2366, Cell Signaling Technology), followed by HRP-conjugated anti-mouse IgG secondary antibody (7076, Cell Signaling Technology). Signal was developed using TMB substrate, and the enzymatic reaction was terminated with stop solution. Absorbance was measured at 450 nm using a microplate reader, and binding activity was quantified based on the resulting absorbance values.

### Cytokine and chemokine analysis

Proinflammatory cytokines and chemokines released by human microglia were measured using ELISA (DY206-05, DY208-05, DY201-05, DY210-05, and DY266-05, R&D Systems), multiplex assay (Luminex Human Magnetic Assay Kit (R&D Systems) and an antibody array (Human Inflammatory Cytokine Antibody Array 5, RayBiotech).

### Intracellular Ca^2+^ imaging

Intracellular calcium levels in SNs were measured using Fluo-4 AM calcium indicator (F10489, Invitrogen) according to the manufacturer’s protocol. Briefly, SNs were loaded with Fluo-4 AM in PowerLoad^™^ solution and incubated at 37 °C for 30 min in the dark, followed by an additional 30 min at room temperature. After washing with Live Cell Imaging Solution (LCIS; A59688DJ, Invitrogen), ESC-SN cultures were treated with conditioned media derived from ESC-MG or HMC3 cultures. Changes in intracellular calcium levels were monitored using a Zeiss LSM900 confocal microscope with standard FITC filter settings (excitation/emission: 494/506 nm). Image sequences were imported into ImageJ (NIH) and regions of interest were selected across the entire field. For visualization, fluorescence intensity colormaps were generated in Fiji using lookup tables with a calibration bar.

### Statistical analysis

Data are presented as mean ± standard deviation (SD) from at least three independent experiments. Statistical analysis was performed using one-way analysis of variance (ANOVA) followed by Tukey’s post hoc test for multiple group comparisons, or Student’s t-test for pairwise comparisons. Statistical significance was defined as *p* < 0.05. All statistical analyses were performed using GraphPad Prism 9 software (GraphPad Software, San Diego, CA, USA).

## Results

### VZV infection in human microglia alters microglial transcriptome

**Fig. 1:**
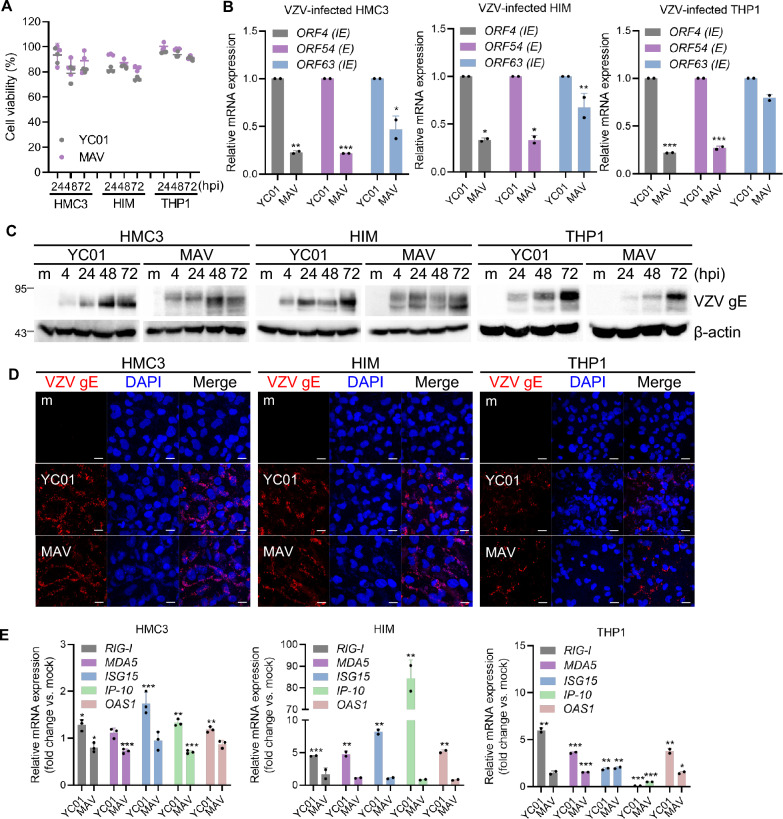
Upregulation of VZV gene and protein expression in human microglial cell lines. **A** HMC3, HIM and THP1 cells were infected with VZV (MOI 0.005) for indicated times and cell viability was measured by CCK-8 assay. Relative cell viability was normalized to mock (m)-infected group and presented as mean ± SD. **B** Cells were infected with VZV for 48 h. VZV *ORF4* (Immediate early gene, IE), *ORF54* (Early gene, E), and *ORF63* (Immediate early gene, IE) expression levels were quantified by RT-qPCR. Data are shown as mean ± SD. **C** Cells were infected with VZV and harvested at 4, 24, 48, or 72 h post-infection (hpi). Cell lysates were subjected to western blotting using antibodies against VZV gE. β-actin was used as a loading control. **D** VZV-infected cells were stained with anti-VZV gE antibody for confocal microscopy at 48 hpi. Scale bar = 20 μm. **E** Cells were infected with VZV for 48 h. The expression levels of *RIG-I*, *MDA5*, *ISG15*, *IP-10*, and *OAS1* were quantified by RT-qPCR, and expressed as fold changes relative to m-infected cells within each cell type. The graphs are representatives of at least 2–3 independent experiments. Statistical analyses were performed using unpaired Student’s t-test or one-way ANOVA followed by Tukey’s post hoc test, as appropriate. *p < 0.05, **p < 0.01, ***p < 0.001 compared to m-infected group

We used various microglial cell culture models to comprehensively analyze microglial gene signatures, phenotypes, and functions in response to cell-free WT strain YC01 or attenuated strain MAV infection. Cell-free YC01 was prepared as described in Fig. S1A, B. To assess the susceptibility of HMC3 microglia to VZV infection, cells were inoculated with cell-free YC01 or MAV. Expression of VZV *ORF63* (immediate-early gene) was quantified via RT-qPCR. Our findings demonstrate that VZV gene and protein expression were found to be upregulated in HMC3 cells (Fig. S1C, D). Next, we compared the magnitude and duration of VZV replication using the existing microglial cell models, HMC3 and HIM. Similar to the human monocytic cell line THP1, both microglial cell lines exhibited minimal cytotoxicity during the early stages of VZV infection (Fig. 1A). Moreover, VZV infection led to comparable expression levels of VZV *ORF4* (immediate-early gene), *ORF54* (early gene), and *ORF63* in all three cell lines (Fig. 1B). YC01 strain appears to have a slightly higher replication efficiency compared to MAV strain in these microglial models. Furthermore, VZV glycoprotein E (gE) protein expression increased in a time-dependent manner upon VZV infection (Fig. 1C, D). Next, we measured the activation status of microglia with phagocytic activity, and analyzed the expression of CD45 and CD68 using flow cytometry. The expression levels of both CD45 and CD68 were markedly elevated in YC01-infected cells compared with MAV-infected cells, indicating robust microglial uptake and activation induced by YC01 (Fig. S2). Given that phagocytosis is a key function of microglia, we investigated whether VZV infection modulates microglial phagocytic activity. YC01 infection enhanced the surface expression of phagocytosis-related molecules, including CD14, CD36, AXL, and TLR2, in both HMC3 and HIM cells (Fig. S3A). Using flow cytometry, we found that VZV infection enhanced latex bead uptake by microglia, with YC01 infection inducing a marked increase in phagocytosis in a multiplicity of infection-dependent manner (Fig. S3B). Functionally, VZV infection promoted microglial phagocytosis of apoptotic cells, with YC01 eliciting a more pronounced effect (Fig. S3C, D).

We conducted bulk RNA sequencing of VZV-infected HMC3 cells to further characterize the microglial response to VZV (Fig. S4). YC01 and MAV induced distinct mRNA expression patterns, whereas the gene profiles from MAV were similar to those of mock-infected cells (Fig. S4A, B). Gene Ontology (GO) enrichment analysis of upregulated differentially expressed genes (DEGs) in YC01-infected HMC3 revealed significant enrichment of antiviral defense pathways, including "defense response to virus" and "response to virus" (Fig. S4C). Consistently, heatmap analysis showed higher expression of immune- and inflammation-related genes in YC01-infected cells than in MAV-infected cells (Fig. S4D). Higher transcript levels of *Retinoic acid-inducible gene I (RIG-I), Melanoma Differentiation-Associated protein 5 (MDA5), IFN-stimulated gene 15 (ISG15), IFN gamma-induced protein 10 kDa (IP-10),* and *2'-5'-Oligoadenylate Synthetase 1 (OAS1)* were detected in YC01-infected cells compared with MAV-infected cells (Fig. 1E). These findings suggested that WT VZV infection, compared with attenuated VZV infection, induced elevated innate immune activation.

**Fig. 2:**
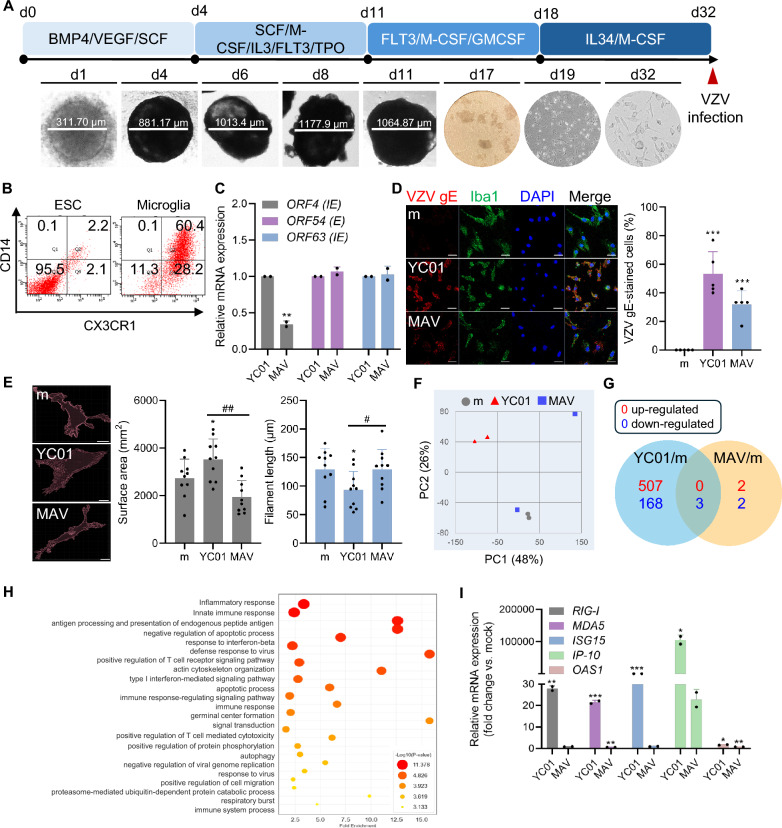
Transcriptomic analysis of human microglia derived from embryonic stem cells reveals distinct innate immune gene signatures. **A** Schematic illustrating the differentiation process of microglia derived from human embryonic stem cells (ESC-MG). **B** Embryonic stem cells (ESCs) and mature microglia were stained with antibodies against CD14 and CX3CR1 and analyzed by flow cytometry. **C** Following the infection of ESC-MG with VZV (MOI 0.01) for 48 h, mRNA levels of VZV *ORF4, ORF54* and *ORF63* were quantified using RT-qPCR. **D** ESC-MG infected with mock (m) or VZV were stained with VZV gE (red) and Iba1 (green) for confocal microscopy. VZV gE-stained cells/total cells were counted using at least 5 images and the graph on the right panel shows % VZV gE-stained cells. Scale bar = 50 μm. **E** ESC-MG infected with m or VZV were stained with Iba1 and microglial morphodynamics was quantified using Imaris software. Scale bar = 10 μm. Data are expressed as mean ± SD (n = 10). **F** Bulk RNA sequencing was performed following the infection of ESC-MG with VZV for 48 h. Principal component analysis of differentially expressed gene (DEG) is shown. **G** Venn diagram showing the number of overlapping and distinct DEGs (adjusted *p* < 0.05, log_2_ fold-change > 0.5) between YC01- and MAV-infected ESC-MG. **H** Gene ontology (GO) enrichment analysis represents the functions of DEGs using circle size and color intensity in the YC01-infected ESC-MG. Larger circles and/or darker colors indicate greater enrichment or statistical significance of a particular GO term within DEGs. **I** Expression levels of *RIG-I*, *MDA5*, *ISG15*, *IP-10*, and *OAS1* were quantified by RT-qPCR in ESC-MG infected with VZV for 48 h. Statistical analyses were performed using unpaired Student’s t-test or one-way ANOVA followed by Tukey’s post hoc test, as appropriate. *p < 0.05, **p < 0.01, ***p < 0.001 compared to the m-infected group. ^#^*p* < 0.05, ^##^*p* < 0.01 compared to the YC01-infected group

ESC-MG serves as a robust model that closely mimics authentic microglia, especially concerning their phenotypes, surface markers, and gene expression profiles [12]. As illustrated in Fig. 2A, ESCs differentiated into microglial progenitor cells, which were then cultured for 14 days to reach maturation. Maturation was confirmed on day 25 by assessing the surface expression of CD14 and CX3CR1, which are primary markers of microglial progenitor cells (Fig. 2B) [30]. To establish VZV infection model in ESC-MG, we first measured the expression of VZV genes (*ORF4*, *ORF54*, and *ORF63*) and gE protein. RT-qPCR confirmed that both YC01 and MAV replicated efficiently in ESC-MG, whereas there were more gE-stained cells in YC01 than in MAV, as detected by immunofluorescence (Fig. 2C, D). Notably, YC01 infection induced distinct morphological changes in ESC-MG, including soma enlargement and a shift to an amoeboid shape, as shown by Iba1 staining. Three-dimensional morphodynamic analysis revealed increases in surface area but a reductions in filament length, specifically in YC01-infected microglia. In contrast, MAV-infected microglia maintained homeostatic morphology without significant structural changes (Fig. 2E).

Analysis of ESC-MG transcriptome via PCA showed that YC01 infection induced a unique molecular signature, sharply contrasting with the overlapping profiles of the mock and MAV-infected groups. While MAV-infected ESC-MGs clustered closely with the mock-infected controls, YC01-infected groups exhibited a profound shift in gene expression characterized by the rapid upregulation of pro-inflammatory and antiviral genes (Fig. 2F–I and Table S2). This highlighted the markedly stronger transcriptional activation induced by YC01, but not by MAV, consistent with the findings in HMC3 cells (Fig. S5). GO analysis of YC01-infected ESC-MG revealed an upregulation of the inflammatory response, innate immune response, and pathways associated with the “defense response to virus” (Fig. 2H), which paralleled the transcriptional signatures observed in HMC3 cells. Moreover, the full list of common microglial state genes described by Krasemann et al. was examined in our dataset [31]. ESC-MG infected with YC01 exhibited a more pronounced upregulation of genes (*APOE, TYROBP,* and *TMEM119*) linked to disease-associated microglia (DAM) phenotype than MAV-infected cells (Fig. S5). KEGG pathway analysis revealed significant upregulation of pathways associated with herpes simplex virus 1 infection, suggesting that YC01 leads to a robust microglial response (Fig. S6). Furthermore, RT-qPCR analysis revealed significantly higher transcript levels of *RIG-I, MDA5, ISG15, IP-10*, and *OAS1* in YC01-infected ESC-MG, whereas these genes were not upregulated in MAV-infected cells (Fig. 2I), This supports the conclusion that WT VZV infection, compared to attenuated VZV infection, induces stronger innate immune activation.

### VZV infection contributes to neuroinflammatory profiles in microglia

Previous studies identified 24 non-synonymous mutations in VZV vaccine strain MAV, 12 of which were located in ORF62 (Fig. S7A) [32]. To determine if these vaccine-specific mutations modulate NF-κB-mediated inflammation, we transiently transfected microglia with either WT or MAV-derived ORF62 mutant plasmids. Because our RNA sequencing data highlighted IP-10 as a key neuroinflammatory chemokine with established NF-κB-responsive elements, we used it as a functional readout. After confirming comparable transfection efficiency of HA-tagged ORF62 in HMC3 cells, we evaluated the effect of ORF62 on NF-κB and IP-10 promoter activity in HMC3 cells (Fig. S7B–E). WT ORF62-expressing cells consistently showed higher promoter activity than mutant ORF62-expressing cells. Next, we examined p65 translocation in HEK293T cells using immunostaining to assess whether ORF62 overexpression induces NF-κB activation. p65 was fully translocated to the nucleus in WT ORF62-expressing cells, whereas only partial translocation was observed in mutant ORF62-expressing cells (Fig. S7F). Additionally, ELISA in WT ORF62-overexpressing HMC3 cells demonstrated significantly elevated secretion of IL-6, IL-8, IL-1β, and TNF-α compared with mutant ORF62-expressing cells in response to LPS stimulation in human microglia (Fig. S7G).

**Fig. 3:**
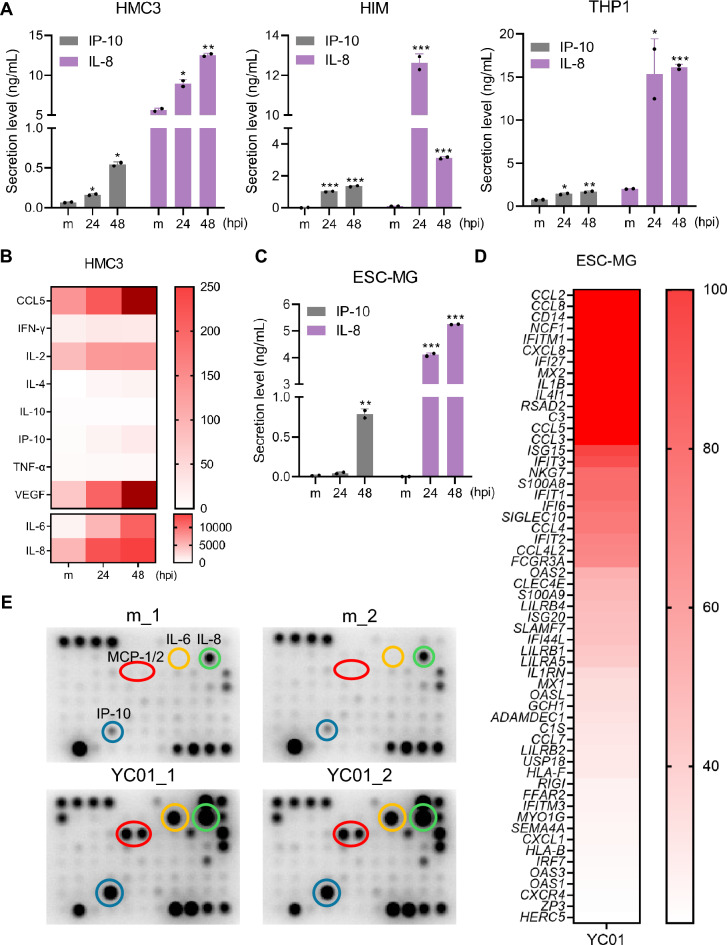
VZV infection induces robust secretion of proinflammatory cytokines in microglia. **A** Cytokine levels from supernatants of mock (m) or VZV (MOI 0.005)-infected HMC3, HIM, and THP1 cells were measured by ELISA. **B** Heatmap representation of cytokine production in supernatants from m or VZV-infected HMC3 with color intensity indicating relative abundance, measured by Luminex multiplex assay. **C** Cytokine levels in supernatants from VZV (MOI 0.01)-infected microglia derived from human embryonic stem cells (ESC-MG) were measured by ELISA. **D** Bulk RNA sequencing was performed on ESC-MG infected with m or VZV for 48 h. Heatmap represents the enrichment of innate immunity or inflammation related genes differentially expressed. **E** Quantification of proinflammatory cytokines in the supernatant of ESC-MG infected with m or VZV for 48 h was measured by human cytokine antibody array. Significant upregulation of proinflammatory cytokines and chemokines such as IL-6, IL-8, IP-10, and MCP-1/2, is shown. Statistical analyses were performed using unpaired Student’s t-test. Data are expressed as mean ± SD. *p < 0.05, **p < 0.01, ***p < 0.001 compared to the m-infected cells

Considering the proinflammatory role of VZV ORF62, we further assessed cytokine and chemokine production in YC01-infected HMC3, HIM, and THP1 cells by analyzing supernatants collected at 24 and 48 h post-infection. YC01 infection led to increased secretion of IP-10 and IL-8 in HMC3 and HIM as well as THP1 (Fig. 3A, B). Consistent with these results, YC01-infected ESC-MG showed a marked upregulation of multiple proinflammatory cytokines (Fig. 3C, D). To validate these transcriptomic results at the protein level, we utilized multiplex antibody arrays, which confirmed a robust proinflammatory profile (Figs. 3E and S8). Taken together, these data demonstrate that YC01 infection triggers a significant dysregulation of the cytokine landscape in microglia.

### Upregulation of MARCO by VZV facilitates viral infection in microglia

**Fig. 4:**
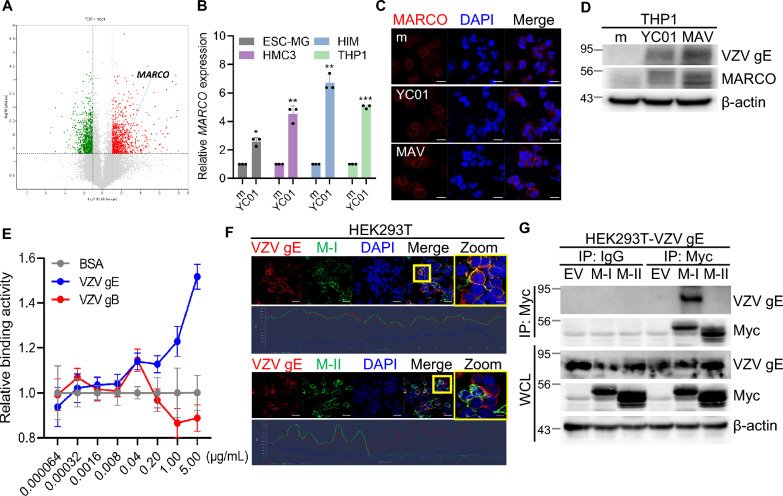
MARCO is upregulated upon VZV infection and interacts with VZV gE. **A** A scatter plot shows that *MARCO* is one of top upregulated genes from YC01-infected ESC-MG. Upregulated (red) and downregulated (green) genes are shown. **B** Relative *MARCO* mRNA expression levels were quantified by RT-qPCR in mock (m)- or YC01-infected cells. ESC-MG cells were infected with YC01 (MOI 0.01), while HIM, HMC3, and THP1 cells were infected with YC01 (MOI 0.005). **C** Immunofluorescence staining of MARCO (red) was performed in THP1 cells infected with m or VZV. Nucleus was stained with DAPI (blue). Scale bar = 20 μm. **D** VZV-infected THP1 cells were harvested at 48 h post-infection. Cell lysates were subjected to western blotting using antibodies against VZV gE and MARCO. β-actin was used as a loading control. **E** Binding affinity between recombinant VZV gE and MARCO was evaluated by ELISA. Plates were coated with increasing concentrations of VZV gE, VZV gB, or bovine serum albumin (BSA) overnight and subsequently incubated with 2 μg/ml of recombinant MARCO protein. Binding activity was measured across a serial dilution range and is expressed as a fold change relative to BSA negative control. **F** Representative confocal images of HEK293T co-transfected with VZV gE and Myc-tagged full-length MARCO (M-I) or scavenger receptor cysteine-rich (SRCR)-deleted MARCO (M-II) plasmids to assess protein co-localization. Qualitative analysis of co-localization between VZV gE (red) and MARCO (green) was performed using ZEN software system. Scale bar = 10 μm. **G** HEK293T cells stably expressing VZV gE (HEK293T-VZV gE) were transfected with Myc-tagged M-I or M-II plasmids. Co-immunoprecipitation and western blotting performed using anti-MYC or anti-VZV gE antibodies to assess the interaction between MARCO and VZV gE. WCL denotes whole cell lysates. All data represent the mean ± SD of at least three independent experiments. Statistical analyses were performed using unpaired Student’s t-test *p < 0.05, **p < 0.01, ***p < 0.001 compared to the m-infected group

Previous reports have suggested that several viruses such as herpes simplex virus 1 (HSV-1), vaccinia virus, and adenovirus exploit MARCO to enhance viral adsorption and infection [33–35]. Although the role of MARCO in VZV infection remains unclear, its established involvement in viral entry and scavenger receptor-mediated responses prompted us to further investigate its potential contribution in this context. Interestingly, *MARCO* was one of the upregulated DEGs in YC01-infected microglia*,* whose expression was markedly upregulated (> sixfold) in YC01-infected ESC-MG (Fig. 4A). RT-qPCR analysis validated the increased *MARCO* mRNA expression induced by VZV infection in all microglia tested, including HMC3, HIM, and ESC-MG (Fig. 4B). Together with microglia, MARCO expression was elevated in VZV-infected THP1 cells than in mock, as determined by immunofluorescence and immunoblotting assays (Fig. 4C, D). Therefore, we investigated the role of MARCO in VZV-infected microglia. Considering that VZV gE is the most abundant protein of VZV and contributes to viral entry by interacting with cellular surface receptors [36], we tested the interaction of VZV gE with MARCO by conducting a binding assay using purified gE and recombinant MARCO, which revealed a dose-dependent interaction between the two proteins, but not with VZV gB, supporting VZV gE-MARCO’s specific interactions (Fig. 4E). Confocal microscopy of HEK293T cells co-transfected with MARCO and gE showed that full-length MARCO (M-I) exhibited strong colocalization with VZV gE at the cell membrane, whereas scavenger receptor cysteine-rich (SRCR)-deleted MARCO (M-II) showed a markedly reduced overlap, indicating the importance of the SRCR domain of MARCO in VZV gE binding (Fig. 4F). Consistently, Co-IP demonstrated a robust interaction between VZV gE and M-I, but not M-II, further supporting the role of the SRCR domain in mediating MARCO-VZV gE interactions (Fig. 4G).

**Fig. 5:**
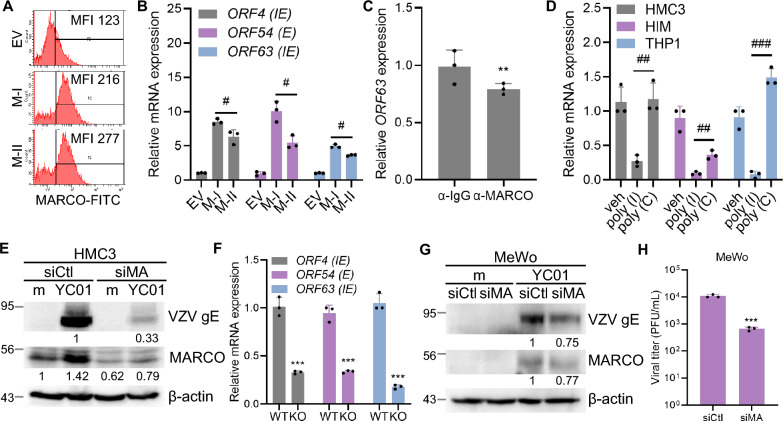
MARCO facilitates VZV replication in microglia. **A** Surface expression of MARCO was analyzed by flow cytometry in HMC3 cells overexpressing empty vector (EV), Myc-tagged full-length MARCO (M-I) or scavenger receptor cysteine-rich (SRCR)-deleted MARCO (M-II) plasmids at 24 h post-transfection. **B** HMC3 cells overexpressing EV, M-I, or M-II were infected with YC01 (MOI 0.005) for 4 h. VZV *ORF4, ORF54* and *ORF63* expression levels were measured by RT-qPCR. **C** HMC3 cells were pre-treated with 10 μg/mL of control IgG (α-IgG) or MARCO blocking antibody (α-MARCO) for 1 h, followed by YC01 infection for 4 h. VZV *ORF63* mRNA was measured by RT-qPCR. **D** Cells were pre-treated with vehicle (veh), poly (I), or poly (C) for 1 h, followed by YC01 (MOI 0.005) infection for 4 h. Expression of VZV *ORF63* was quantified by RT-qPCR. **E** HMC3 cells were transfected with control (siCtl) or MARCO (siMA)-specific siRNA. After 30 h, cells were infected with YC01 for 24 h, and immunoblotting was performed to detect MARCO and VZV gE protein expression. β-actin was used as a loading control. **F** Wild-type (WT) and MARCO-knockout (KO) HMC3 cells were infected with YC01 for 4 h. The expression levels of VZV *ORF4, ORF54,* and *ORF63* were quantified by RT-qPCR. **G**, **H** MeWo cells were transfected with siCtl or siMA and infected with YC01. Viral quantification was performed by immunoblotting (G) and plaque assay (H). Statistical analyses were performed using unpaired Student’s t-test or one-way ANOVA followed by Tukey’s post hoc test, as appropriate. All data represent the mean ± SD of at least three independent experiments. Statistical significance *p < 0.05, **p < 0.01, ***p < 0.001 compared to EV-, α-IgG-, Veh-, WT-, or siCtl-treated group. ^#^*p* < 0.05, ^##^*p* < 0.01, ^###^*p* < 0.001 compared to M-I or poly (I)-treated group

We overexpressed either the M-I- or M-II plasmids in HMC3 cells to further investigate the role of MARCO in VZV replication, confirming more than twofold increase in surface MARCO levels using flow cytometry (Fig. 5A). Cells overexpressing M-I exhibited significantly elevated expression of VZV *ORF4*, *ORF54*, and *ORF63* upon YC01 infection compared with those containing the empty vector. Notably, M-II failed to enhance viral gene expression to the same extent as the SRCR-containing M-I, suggesting a functional requirement for this domain (Fig. 5B).

Next, we used a MARCO-blocking antibody and ligands for the class A scavenger receptor poly(I), which is known to block HSV-1 adsorption to keratinocytes by inhibiting MARCO function [33]. Treatment with the blocking antibody or ligands significantly suppressed VZV gene expression compared to that in control IgG-, vehicle-, or poly(C)-treated cells (Fig. 5C, D). Furthermore, MARCO-specific siRNA treatment in HMC3 cells led to a ~ 70% decrease in VZV gE protein levels, as determined by immunoblotting (Fig. 5E). To assess the role of MARCO in early VZV replication, WT and MARCO-knockout (KO) HMC3 cells were generated and infected with YC01 for 4 h, and viral gene expression was evaluated using RT-qPCR. Transcription of *ORF4*, *ORF54*, and *ORF63* was significantly reduced in MARCO-deficient cells compared to WT HMC3 cells (Fig. 5F). To confirm this data, we performed a immunoblotting and plaque assay to evaluate the effect of MARCO expression on VZV titers. Knockdown of MARCO in MeWo cells using siRNA resulted in a reduction in viral titers and viral protein expression compared to control siRNA-treated cells (Fig. 5G, H). These data indicate that MARCO significantly promotes VZV replication, regardless of the cell type.

### VZV enhances TLR2/MARCO-mediated microglial inflammation

**Fig. 6:**
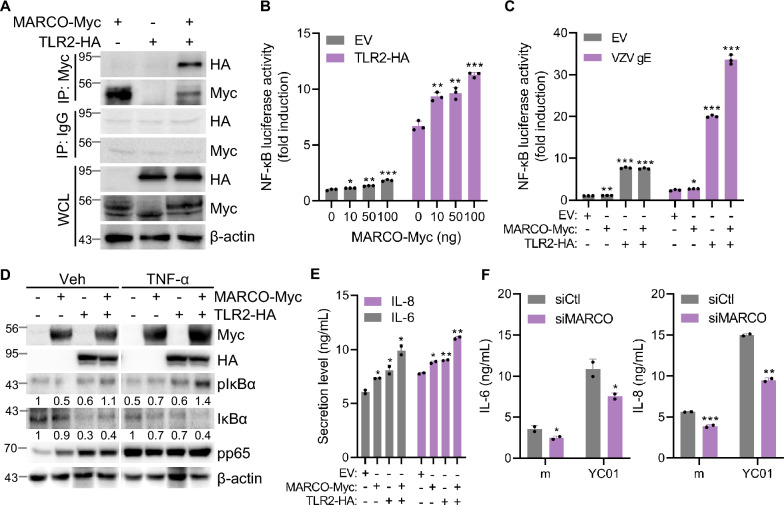
MARCO enhances proinflammatory cytokine production in VZV-infected microglia. **A** HEK293T cells were co-transfected with Myc-tagged full-length MARCO and HA-tagged TLR2 plasmids. Cell lysates were subjected to co-immunoprecipitation using anti-Myc antibodies to evaluate the interaction between MARCO and TLR2. WCL denotes whole cell lysates. **B** HEK293T cells were co-transfected with empty vector (EV) or HA-tagged TLR2 and increasing amounts of Myc-tagged MARCO plasmid. Relative NF-κB luciferase activity was measured. **C** HEK293T cells were transfected with VZV gE (100 ng) along with either EV, Myc-tagged MARCO, or HA-tagged TLR2 plasmids. NF-κB luciferase activity was measured. **D** HEK293T cells were transfected with Myc-tagged MARCO and/or HA-tagged TLR2 expression plasmids as indicated and treated with vehicle (Veh) or TNF-α. Cell lysates were harvested 24 h post-transfection and subjected to immunoblot analysis using antibodies against phospho-IκBα (pIκBα), total IκBα (IκBα), and phospho-p65 (pp65) to assess the activation of NF-κB signaling pathway. Numbers below the blot represent quantification by densitometric analysis. β-actin was used as a loading control. **E** HMC3 cells were transfected with EV, Myc-tagged MARCO, or HA-tagged TLR2 plasmids for 24 h, followed by YC01 infection (MOI 0.005) for 24 h. IL-6 and IL-8 levels in the cell culture supernatants were measured by ELISA. **F** HMC3 cells were transfected with control-(siCtl) or MARCO-specific siRNA (siMARCO) for 30 h, followed by infection with mock (m) or YC01 for 24 h. IL-6 and IL-8 levels in the supernatant were quantified by ELISA. Statistical analyses were performed using unpaired Student’s t-test or one-way ANOVA followed by Tukey’s post hoc test, as appropriate. *p < 0.05, **p < 0.01, ***p < 0.001 compared to the EV-, or siCtl-transfected-m group

VZV is known to trigger TLR2-mediated NF-κB activation and SRCR domains of MARCO enhance NF-κB activity via TLR2/CD14 [26, 37]. As previously described, we confirmed the direct interaction between full-length MARCO and TLR2 using Co-IP assays (Fig. 6A) [38]. We performed a luciferase reporter assay in HEK293T cells to investigate whether MARCO modulates NF-κB signaling downstream of TLR2. Increasing amounts of MARCO co-transfected with TLR2 led to a dose-dependent enhancement of NF-κB promoter activity, whereas MARCO alone induced only minimal activation, indicating a synergistic role of MARCO in TLR2-mediated signaling (Fig. 6B). Furthermore, co-expression of MARCO and TLR2 significantly enhanced VZV gE- or TNF-α-induced NF-κB activation compared to either molecule alone (Fig. 6C, D). TLR2 is a key player in the innate immune system, and its activation triggers a cascade that prominently involves NF-κB signaling and cytokine production; thus, we examined the immunomodulatory role of MARCO and TLR2 during VZV infection in microglia. Notably, MARCO and TLR2 overexpression triggered increased levels of proinflammatory cytokines, such as IL-6 and IL-8, whereas blocking MARCO by siRNA transfection led to the opposite effect in VZV-infected human microglia (Fig. 6E, F). These results suggest that VZV enhances TLR2/MARCO-mediated microglial inflammation.

### VZV-induced microglial cytokine production amplifies sensory neuron excitability

VZV exhibits neurotropism and preferentially establishes latency in SN [39, 40]. Human neuroblastoma-derived SH-SY5Y cells are increasingly used as models to study VZV infections in neurons [41, 42]. To underscore the role of VZV-induced microglia-mediated neuroinflammation in neuronal vulnerability, we exposed SH-SY5Y cells to conditioned medium (CM) derived from mock- or VZV-infected microglia (Fig. S9A). Treatment of neuronal cells with CM from VZV-infected microglia increased the number of PI^+^ and DCF-DA^+^ cells, indicating VZV-induced cell death and ROS production (Fig. S9B). Consistent with neuronal death, the number of degenerating SH-SY5Y labeled with Fluoro-Jade C dye was elevated in SH-SY5Y exposed to CM from VZV-infected microglia (Fig. S9C).

**Fig. 7:**
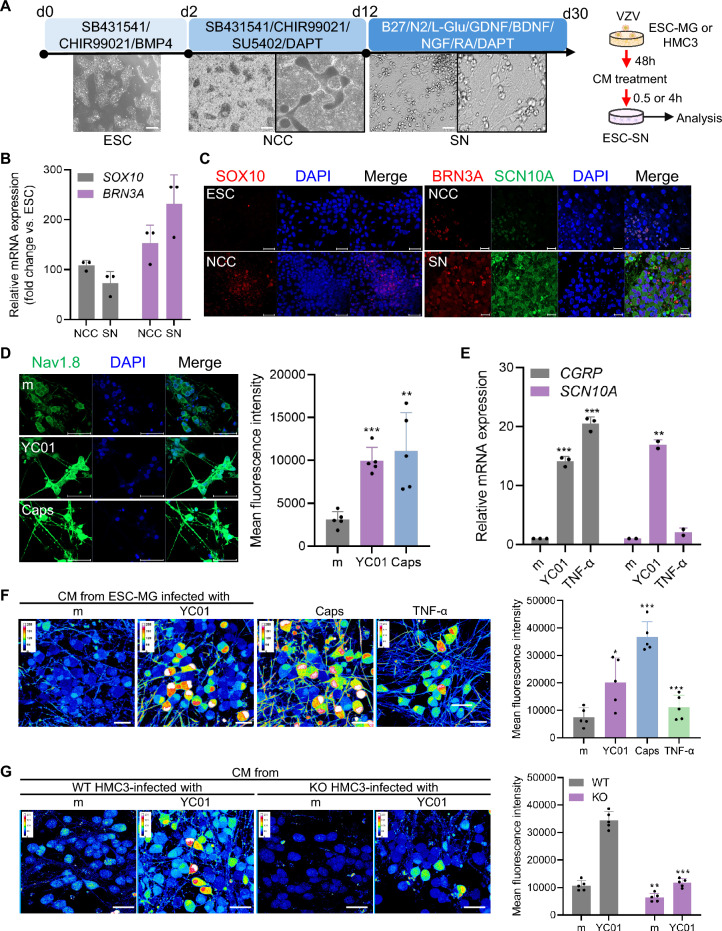
Secretome of VZV-infected microglia upregulates spontaneous sensory neuron activity. **A** A schematic representation of the differentiation protocol to generate sensory neurons (SNs) from human embryonic stem cells (ESC-SN). A box represents the magnified area of an image. Scale bar = 400 μm. **B** Expression levels of neural crest cells (NCC) markers, *SOX10* and *BRN3A,* were quantified by RT-qPCR in human embryonic stem cells (ESCs, day 2), NCCs (day 12), and SNs (day 30). **C** Immunofluorescence images reveal properly differentiated ESC, NCC, and SN. ESC and NCC were stained for SOX10 (red) and DAPI (blue). Scale bar = 50 μm. NCC and SN were stained for BRN3A (red), SCN10A (green), and DAPI (blue). Scale bar = 20 μm. **D** ESC-SN were treated with conditioned medium (CM) from mock (m)- or YC01 (MOI 0.005)-infected HMC3 cells for 4 h. Nav1.8 expression was examined by immunofluorescence staining (green) with DAPI (blue). Scale bar = 50 μm. **E** Expression levels of *CGRP* and *SCN10A* were quantified by RT-qPCR. **F**, **G** ESC-SN were treated with CM from m- or YC01 (MOI 0.01)-infected microglia derived from human embryonic stem cell (ESC-MG) or directly treated with 1 μM Capsaicin (Caps) or 50 ng/mL TNF-α (**F**). ESC-SN were treated with CM from MARCO wildtype (WT) or knockout (KO) HMC3. Calcium signals were recorded using Fluo-4 AM dye (**G**). Scale bar = 20 μm. Statistical analyses were performed using unpaired Student’s t-test or one-way ANOVA followed by Tukey’s post hoc test, as appropriate. *p < 0.05, **p < 0.01, ***p < 0.001 compared to ESC-, or m-treated group

ESC-SN mimic the function of DRG neurons in detecting pain, temperature, and touch [43].Therefore, we investigated the effect of the microglial secretome from VZV-infected cells on the ESC-SN. Notably, ESC-SN were differentiated and cultured at high purity using optimized induction protocols involving SMAD inhibition, Wnt signaling activation, and SHH signaling modulation (Fig. 7A) [19]. We examined the time-dependent expression of the neural crest marker *SOX10* and the nociceptive SN marker *BRN3A* in ESCs, neural crest cells (NCCs), and SNs using RT-qPCR and immunofluorescence staining to validate ESC-SN differentiation (Fig. 7B, C). Of note, mature SNs expressed Nav1.8, a well-known nociceptor-specific sodium channel encoded by *sodium voltage-gated channel alpha subunit 10 (SCN10A)*, which was not detected in NCCs. Immunofluorescence analysis revealed that Nav1.8 expression was markedly increased in ESC-SN treated with CM from YC01-infected HMC3 cells. Notably, a similar upregulation of Nav1.8 was observed following capsaicin treatment (Fig. 7D).

A recent study shows that TNF-α, elevated in VZV-infected microglia, triggered a dose-dependent calcium influx in DRG-derived cells, suggesting a mechanism for inflammatory cytokines-derived pain signaling [44]. To investigate the effect of VZV-infected microglial secretome on ESC-SN responses, the expression levels of major nociceptor markers were measured. In particular, *calcitonin gene-related peptide (CGRP)*, a neuropeptide released by nociceptors that plays a key role in pain signaling and inflammation, and *SCN10A* showed increased mRNA expression in response to treatment with the secretome of YC01-infected microglia (Fig. 7E). In parallel with elevated nociceptor expression, treatment of ESC-SNs with the secretome from YC01-infected microglia resulted in increased Fluo-4 AM fluorescence intensity, a widely recognized indicator of calcium influx and nociceptor activation. These findings suggest that the microglial secretome enhances pain sensitivity and promotes nociceptive signaling in SNs (Fig. 7F). To further examine the role of MARCO in nociceptor activation, ESC-SN were treated for 30 min with CM derived from YC01-infected MARCO WT or KO HMC3 cells. Interestingly, calcium imaging further demonstrated that MARCO WT-derived CM elicited a robust calcium influx, while MARCO KO-derived CM induced only a modest increase, suggesting that the presence of MARCO promotes an upregulation of factors associated with neuropathic pain following VZV infection (Fig. 7G).

## Discussion

Growing evidence has highlighted a multifaceted role of microglia during viral encephalitis and neurodegeneration [45, 46]. In this study, we extended our previous research on VZV-specific host immune responses in human dermal fibroblasts [47], incorporating new insights into microglia to explore the impact of VZV infection on brain immunity. Herein, our results demonstrate for the first time that microglia are susceptible to VZV infection and support viral gene and protein expression, and identify microglial MARCO as an important factor facilitating VZV uptake, as well as modulating TLR2-mediated neuroinflammation and neuronal calcium signaling in ESC-SN. Our study is the first to reveal that VZV infection leads to morphological and functional alterations in microglia.

Previous studies have focused on the characterization of VZV infection in neuronal models, including ESC-derived neurons, neurospheres, human SN, and mixed human neuron-astrocyte 2D neurospheroids [15, 16, 39, 40, 48, 49]. Although recent studies have revealed that VZV can establish infection in non-neuronal cells, such as astrocytes, triggering morphological dysfunction [50–52], the role of microglia during VZV infection remains unknown. Microglia are highly heterogeneous and dynamic cell populations that undergo morphological and phenotypic changes upon stimulation [53]. Upon VZV infection, microglia underwent a phenotypic shift from a homeostatic state to a DAM-like phenotype, characterized by elevated inflammatory profiles. Together with neuroinflammation, VZV-infected microglia were highly capable of robust phagocytosis and dynamic morphological changes, including the removal of apoptotic neuroblastoma cells. It is highly likely that VZV strongly induced the production of inflammatory cytokines in microglia via ORF62, which encodes an immediate-early 62 protein [3–6]. However, studies providing insights into the neurovirulence factors of VZV are limited. Considering that a proinflammatory environment is observed in the spinal cord and cerebrospinal fluid of patients with VZV myelopathy, it would be interesting to further investigate the role of microglial activation in VZV encephalitis model.

One of the highlights of our study is that microglial MARCO, a class A scavenger receptor, can be exploited by VZV to promote viral replication. While MARCO is traditionally recognized for its role in bacterial defense [54], mounting evidence suggests it also functions as a proviral factor. Previous studies have shown that MARCO facilitates the entry or replication of DNA viruses, such as vaccinia and adenovirus [33, 34, 55]. In particular, MacLeod et al. showed that MARCO facilitates HSV-1 infection by interactions with viral glycoprotein C. Our current study builds on this finding, showing that VZV upregulates MARCO expression in microglia, and that MARCO binds to VZV gE through C-terminal SRCR domain to facilitate viral entry and replication. Besides promoting adsorption and infection, MARCO can be involved in host innate immune responses by interacting with TLR2 [38, 54, 56]. TLR2 is traditionally known to recognize bacterial components but also plays an important role in viral recognition [57, 58]. Notably, Wang et al. highlighted that TLR2 and MARCO interact via the SRCR domain in microglia-induced neuroinflammation [38]. In this study, we provide insights into the involvement of MARCO in TLR2-mediated neuroinflammation during VZV infection. We found that MARCO is physically associated with TLR2 and that VZV gE-bound MARCO contributes to TLR2-mediated inflammation. Therefore, it would be interesting to investigate the role of MARCO in neuroinflammation by triggering MARCO and TLR2 interactions using an in vivo model.

VZV infection is followed by an established lifelong latency in the SNs of dorsal root and trigeminal ganglia [1]. Reactivation of VZV can lead to neuronal cell death and persistent post-herpetic neuralgia, a debilitating condition characterized by chronic neuropathic pain, in elderly individuals [1]. Our data demonstrate that the secretome from VZV-infected microglia induces ROS production and degeneration-associated phenotype in neuroblastoma cells. Furthermore, *CGRP* and Na_v_1.8 nociceptor expression were significantly increased in SNs, highlighting the multiple effects of VZV-triggered microgliosis on neuronal cells. Microglia-mediated inflammation and type I IFN responses play pivotal roles in modulating nociceptor expression in SNs, resulting in severe pain responses [59–61]. As shown by our data, VZV-infected microglia significantly released proinflammatory cytokines, including TNF-α, which is known to trigger inflammatory pain responses in mice [62]. It is also highly possible that IFNs produced by VZV-infected microglia binds to IFN-α/β receptor on SNs, altering nociceptor gene expression, and enhancing nociceptive signaling. Furthermore, VZV is detected by cGAS/STING pathway [20]. The subsequent activation of this axis may contribute to pro-inflammatory milieu which could affect neuronal viability. Given that STING activation can exacerbate pain responses in various diseases, such as cancer and low back pain [63–65], it would be interesting to further analyze the composition of the secretome of VZV-infected microglia to uncover the molecular factors contributing to neuropathic pain.

Several studies have reported the potential association between shingles vaccination and a reduced risk of neurodegenerative diseases, yet the exact mechanism remains to be fully investigated [66–68]. Furthermore, VZV vaccines are not included in the vaccination programs of many countries, leading to a continued incidence of VZV-associated diseases, such as shingles and post-herpetic neuralgia. Although VZV infection does not appear to directly trigger amyloid β and tau pathology in neural stem cells, it may contribute to neurodegeneration through VZV-induced gliosis and the subsequent release of inflammatory cytokines, which could facilitate viral reactivation. Notably, in contrast to neural stem cells, VZV-infected primary human spinal astrocytes exhibit elevated levels of amylin and amyloid β aggregates [52]. Importantly, VZV gB may play a role in this process, as it contains amyloidogenic sequences that could promote aggregation [52]. In support of this, recent cohort studies have highlighted that the administration of recombinant shingles vaccine reduces the risk of developing dementia, emphasizing VZV’s contribution to neurodegenerative processes [66, 67]. Considering that our data provide novel insights into the contribution of VZV-induced gliosis and neurodegeneration, it would be meaningful to further investigate how VZV-infected microglia directly or indirectly affect amyloid β and tau pathology to better understand neuropathological changes and dysfunctions caused by VZV.

## Conclusion

In summary, our findings provide novel insights into the neuropathic mechanisms of VZV by demonstrating that human microglia are both susceptible to infection and active drivers of the neuroinflammatory response. Importantly, we characterized a unique mechanism involving the interaction between VZV gE and MARCO, which amplifies TLR2-mediated inflammation in microglia. These findings expand our understanding of VZV-host interactions in the brain and highlight microglia as a potential therapeutic target for mitigating VZV-induced neuroinflammation and associated neurological complications.

## Supplementary Information

Supplementary material 1.

## Data Availability

The datasets generated during the current study are available in the GEO repository under accession number GSE309260.
