# Supplementary material for: Microglial MARCO facilitates Varicella zoster virus uptake and triggers TLR2-mediated neuroinflammation
Source: J Biomed Sci. 2026 May 23;33:53. doi: 10.1186/s12929-026-01256-9 (PMC13198062; doi:10.1186/s12929-026-01256-9)
Supplement: Supplementary file 1 — Supplementary material 1. [file 12929_2026_1256_MOESM1_ESM.docx]

Microglial MARCO facilitates Varicella Zoster Virus replication and triggers TLR2-Mediated neuroinflammation

Ji-Soo Lim^1*^, Soo-Jin Oh^1*^, Ji-Yeun Hur^2^, Subin Oh^3^, Seuk-Min Ryu^4^, Rafael T. Han^3,5^, Hosun Park^6^, Dawn M E Bowdish^7^, Ok Sarah Shin^1,8 #^

^1^ BK21 Graduate Program, Department of Biomedical Sciences, Korea University College of Medicine, Seoul, Korea

^2^ Department of Biological Sciences, Ajou University, Suwon, Korea

^3^ Biomedical Research Division, Korea Institute of Science and Technology (KIST), Seoul, Korea

^4^ Center for Advanced Biomolecular Recognition, Biomedical Research Division, Korea Institute of Science and Technology (KIST), Seoul, Korea

^5^ KHU-KIST Department of Converging Science and Technology, Kyung Hee University, Seoul, Korea

^6^ Department of Microbiology, College of Medicine, Yeungnam University, Daegu, Korea

^7^ Department of Medicine, McMaster University, Hamilton, ON, Canada.

^8^  Vaccine innovation center, Korea University College of Medicine, Seoul, Korea

*Co-first authors

# correspondence: oshin@korea.ac.kr (O.S.S.); Tel.: 82-2-2626-3280

**Conflict of Interest**

The authors declare that this study was conducted in the absence of any commercial or financial relationships that could be construed as potential conflicts of interest.

**Table S1. Primer sequences**

| **Gene name** | **Forward primer** | **Reverse primer** |
| --- | --- | --- |
| ***VZV ORF4*** | GCCCATGAATCACCCTC | ACTCGGTACGCCATTTAG |
| ***VZV ORF54*** | TCCAACCCCTCTTCGGCTCG | GGGGATGGCCGATGGGATGT |
| ***VZV ORF63*** | CCGACGCGGAATCATCGGAC | TGTTGCACCCATCCCCGTCT |
| ***RIG-I*** | GGACGTGGCAAAACAAATCAG | GCAATGTCAATGCCTTCATCA |
| ***MDA5*** | AGGAGTCAAAGCCCACCATCTG | ATTGGTGACGAGACCATAACGGATA |
| ***ISG15*** | GAGAGGCAGCGAACTCATCT | CTTCAGCTCTGACACCGACA |
| ***IP-10*** | CGATTCTGATTTGCTGCCTT | CATTTCCTTGCTAACTGCTTTC |
| ***OAS1*** | TGTCCAAGGTGGTAAAGGGTG | CCGGCGATTTAACTGATCCTG |
| ***MARCO*** | TGTCCGTCAGGATTGTCGG | CTCGTCATCGCAAATTGTCCC |
| ***SOX10*** | CCAGGCCCACTACAAGAGC | CTCTGGCCTGAGGGGTGC |
| ***BRN3A*** | AGTACCCGTCGCTGCACTCCA | TTGCCCTGGGACACGGCGATG |
| ***CGRP*** | TCATTGCCCAGAAGAGAGCC | CAAAGTTGTTCTTCACCACACC |
| ***SCN10A*** | CTGTCGATGTCTCGGCATTC | TGGGCACTTCTGTTCAGACTC |
| ***GAPDH*** | GAAGGTCGGAGTCAACGGATTT | GAATTTGCCATGGGTGGAAT |

**Table S2. List of top 30 differentially expressed genes (DEGs) from VZV-infected ESC-MG**

| **Rank** | **Up-regulated DEGs (Fold change)** | | | |
| --- | --- | --- | --- | --- |
|  | **Gene Symbol** | **YC01 /mock** | **MAV /mock** | **MAV /YC01** |
| **1** | ***CCL2*** | 267.304 | 1.969 | 0.007 |
| **2** | ***CCL8*** | 264.061 | 1.000 | 0.004 |
| **3** | ***CD14*** | 253.058 | 2.436 | 0.010 |
| **4** | ***NCF1*** | 231.438 | 1.222 | 0.005 |
| **5** | ***NCF1C*** | 214.108 | 0.741 | 0.003 |
| **6** | ***IFITM1*** | 209.602 | 14.426 | 0.069 |
| **7** | ***CXCL8*** | 209.586 | 1.278 | 0.006 |
| **8** | ***IFI27*** | 206.641 | 7.216 | 0.035 |
| **9** | ***NCF1B*** | 163.840 | 1.719 | 0.010 |
| **10** | ***PTGES*** | 149.894 | 1.339 | 0.009 |
| **11** | ***MX2*** | 131.632 | 12.507 | 0.095 |
| **12** | ***IL1B*** | 115.793 | 1.003 | 0.009 |
| **13** | ***IL4I1*** | 115.377 | 2.252 | 0.020 |
| **14** | ***SERPINA1*** | 110.138 | 0.890 | 0.008 |
| **15** | ***RSAD2*** | 108.957 | 2.274 | 0.021 |
| **16** | ***C3*** | 105.813 | 2.777 | 0.026 |
| **17** | ***CCL5*** | 103.727 | 1.243 | 0.012 |
| **18** | ***CCL3*** | 102.923 | 2.022 | 0.020 |
| **19** | ***C15orf48*** | 102.722 | 2.194 | 0.021 |
| **20** | ***SRGN*** | 102.563 | 2.240 | 0.022 |
| **21** | ***ISG15*** | 98.166 | 10.339 | 0.105 |
| **22** | ***IFIT3*** | 93.388 | 6.199 | 0.066 |
| **23** | ***NKG7*** | 81.460 | 1.408 | 0.017 |
| **24** | ***S100A8*** | 81.195 | 1.105 | 0.014 |
| **25** | ***IFIT1*** | 81.174 | 4.743 | 0.058 |
| **26** | ***IFI6*** | 77.883 | 22.930 | 0.294 |
| **27** | ***SIGLEC10*** | 76.170 | 1.034 | 0.014 |
| **28** | ***CCL4*** | 75.761 | 0.738 | 0.010 |
| **29** | ***IFIT2*** | 71.680 | 3.018 | 0.042 |
| **30** | ***CCL4L2*** | 70.675 | 1.140 | 0.016 |

**
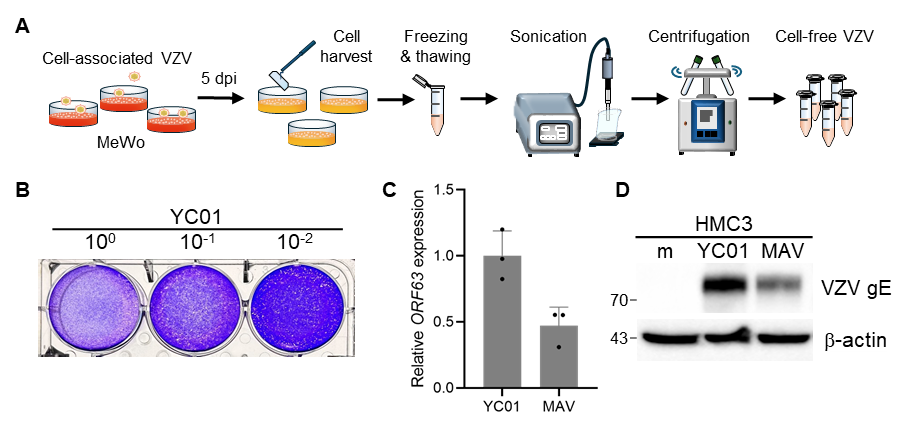
**

**Figure S1. Cell-free VZV preparation procedure**

**A** A schematic diagram depicting the multi-step protocol designed to collect cell-free VZV stocks. **B** A plaque assay was performed to quantify cell-free YC01, yielding a titer of 5×10⁵ PFU/mL. **C** HMC3 were infected with VZV (MOI 0.005, 48 hpi) and VZV ORF63 transcript levels were quantified by RT-qPCR. **D** VZV gE protein expression in HMC3 cells infected with mock (m) or VZV (MOI 0.005, 48 hpi) was assessed by immunoblotting.

**
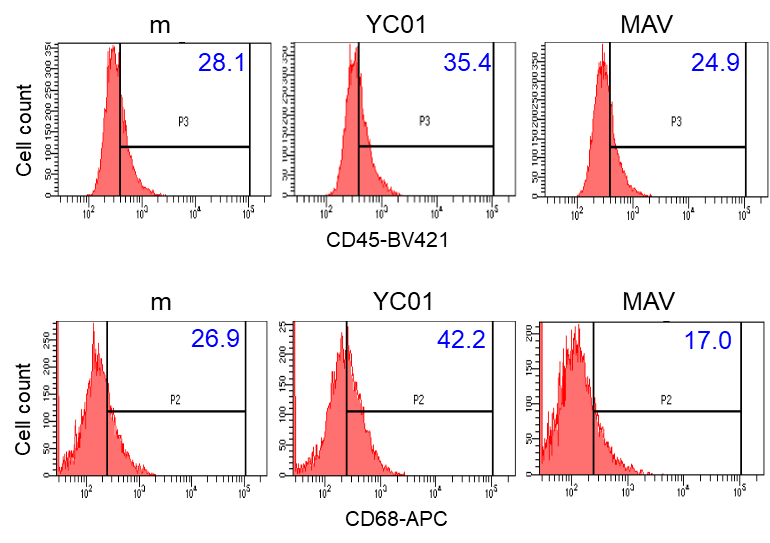
**

**Figure S2. Flow cytometry analysis of CD45 and CD68 in VZV-infected microglia**

HMC3 cells were infected with mock (m), or VZV (MOI 0.005, 48 hpi) for 48 h. Cells were then stained for surface CD45 and intracellular CD68 and expression was analyzed by flow cytometry.


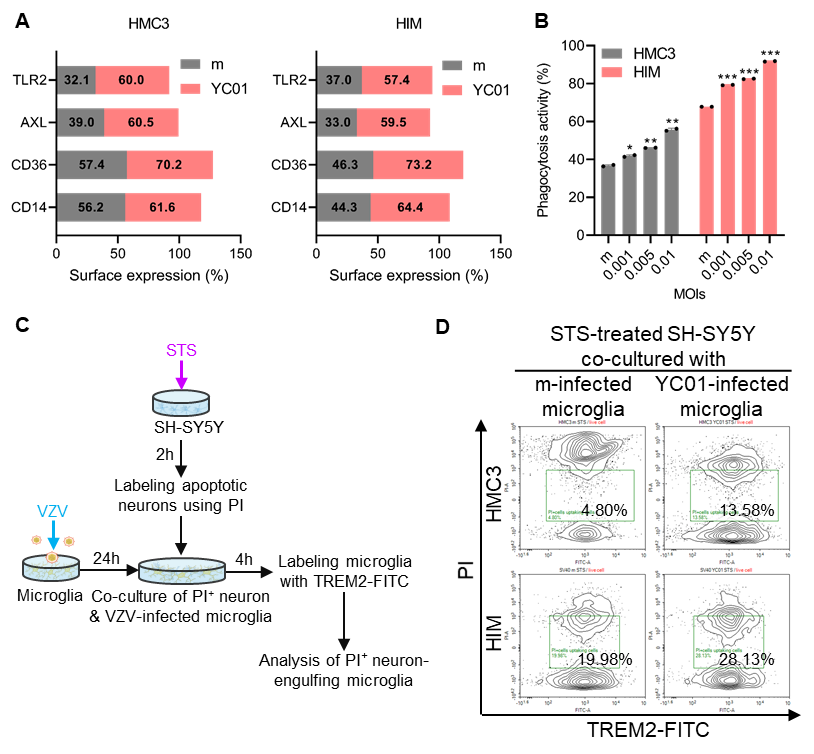


**Figure S3. Enhancement of microglial phagocytic activity by VZV infection**

**A** Surface expression levels of phagocytic receptors, including TLR2, AXL, CD36, and CD14 were analyzed in HMC3 or HIM cells following infection with mock (m) or VZV (MOI 0.005). Representative histograms from flow cytometry analysis are shown. Each result is representative of at least two independent experiments. **B** HMC3 or HIM cells were infected with VZV at indicated MOI for 24 h. Cells were incubated with fluorescent microspheres (6 µm) for 1.5 h, and phagocytic activity was assessed via flow cytometry. **C** Schematic overview of the experimental workflow: Microglia (HMC3 and HIM cells) were infected with mock or VZV (MOI 0.01) for 24 h. SH-SY5Y cells were treated with staurosporine (STS) for 2 hours first and labeled with propidium iodide (PI). PI^+^ SH-SY5Y cells were co-cultured with VZV-infected microglia, followed by staining with anti-TREM2 antibody. Flow cytometry was performed to assess the phagocytic uptake of apoptotic neurons by TREM2-expressing microglial cells. **D** Contour plots show TREM2-FITC versus PI staining, with the gated region indicating PI-positive apoptotic cells within TREM2-expressing microglial populations.

**
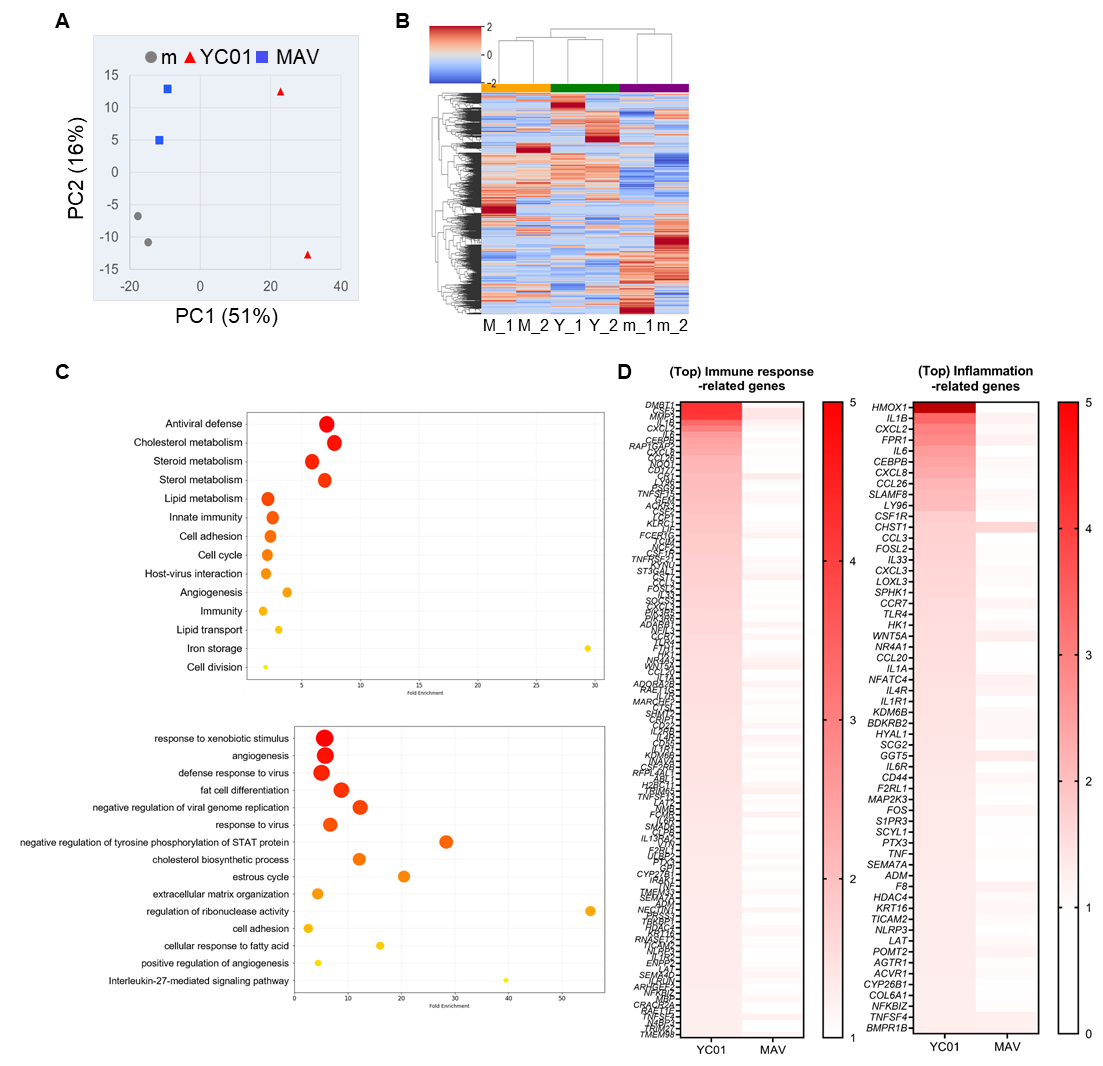
**

**Figure S4. Transcriptomic profiles of VZV-infected HMC3**

**A** HMC3 cells were mock (m)-infected or infected with YC01 (Y) or MAV (M) (MOI 0.005, 48 hpi). Total RNA was extracted and subjected to bulk RNA sequencing. Principal component analysis was applied to score sample variability. **B** Hierarchical clustering heatmap showing differentially expressed genes (DEGs) across all groups, with distinct clustering patterns observed. **C** Dot plots of Gene Ontology (GO) enrichment analysis illustrating significantly enriched biological processes related to innate immune responses and cellular functions following YC01 infection. The x-axis indicates the number of DEGs involved in each term, while the color gradient reflects enrichment significance (-log₁₀ adjusted p-values). **D** Heatmaps showing the expression of DEGs in the category of immune response-related genes (left) and inflammation-related genes (right).


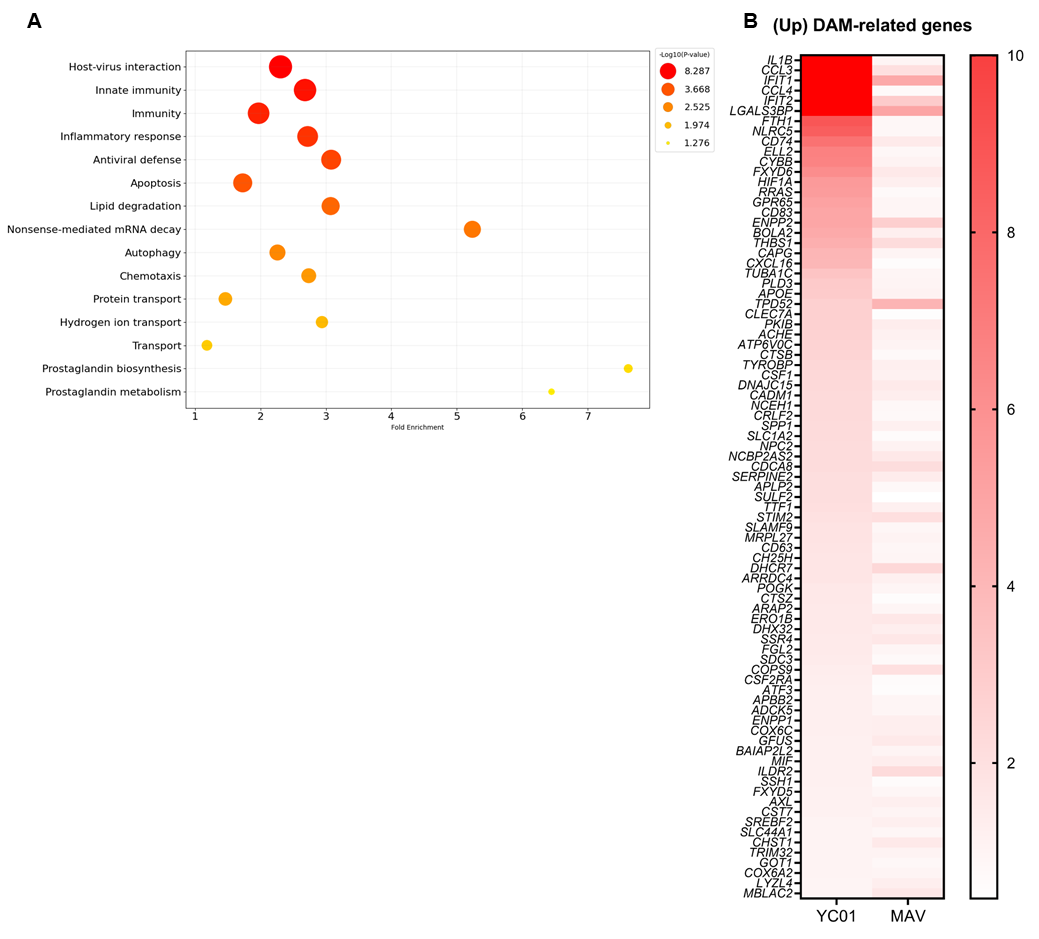


**Figure S5. VZV infection-induced transcriptomic alterations in ESC-MG**

**A** Gene Ontology (GO) enrichment analysis of differentially expressed genes (DEGs) from human embryonic stem cell-derived microglia (ESC-MG) infected with YC01 or MAV (MOI 0.01, 48 hpi), compared with mock-infected controls. The plot shows significantly enriched biological processes associated with innate immune responses and cellular functions after YC01 infection. The y-axis indicates GO terms, the x-axis indicates the number of DEGs involved, and the color scale represents enrichment significance (-log₁₀ adjusted p-values). **B** The expression of disease-associated microglia (DAM)-related genes in ESC-MG are visualized by heatmap analysis. Gene expression was visualized using z-score normalized log₂-transformed counts from bulk RNA sequencing.


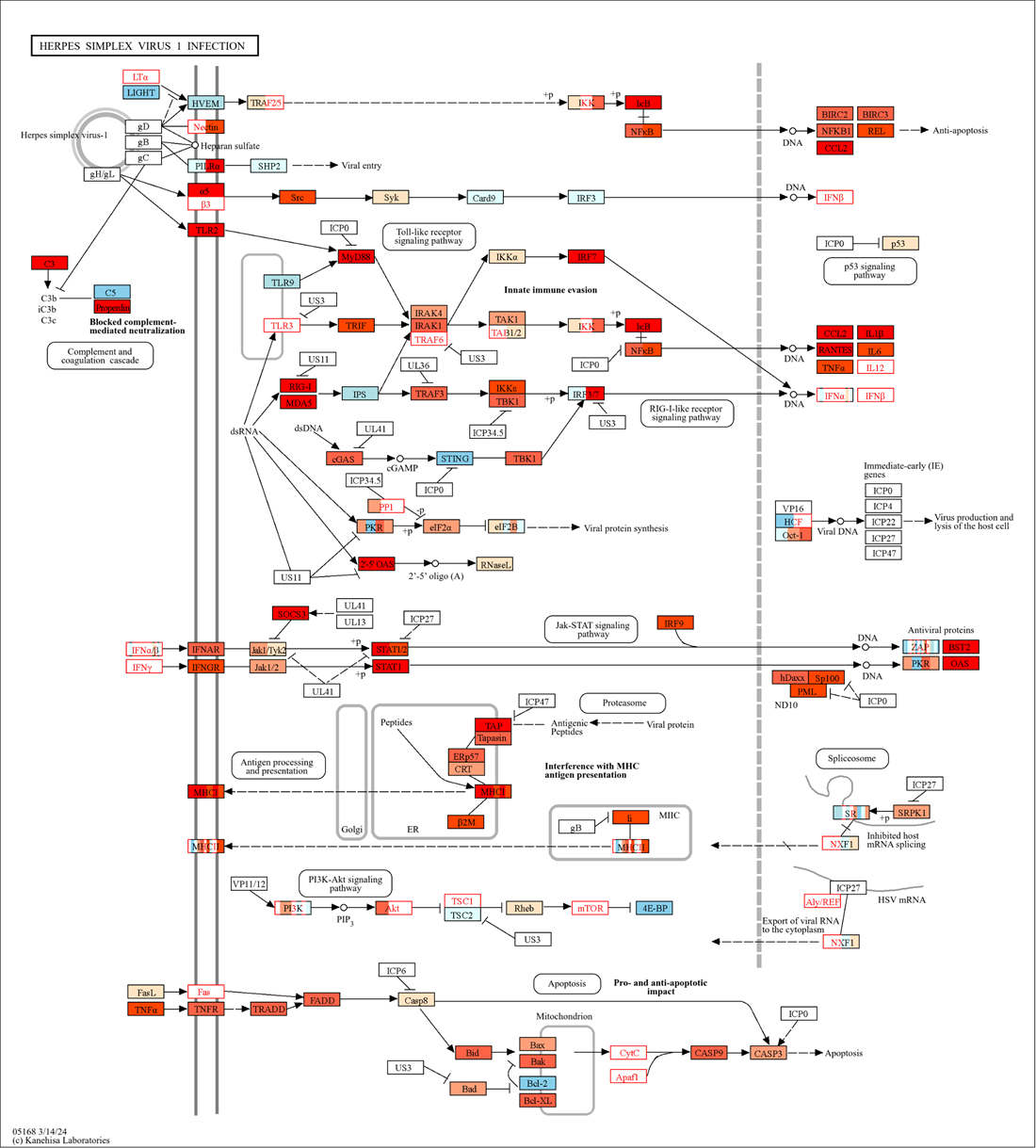
 **Figure S6. KEGG pathway analysis of YC01-infected ESC-MG**

Kyoto Encyclopedia of Genes and Genomes (KEGG) pathway enrichment analysis of differentially expressed genes in YC01-infected human embryonic stem cell-derived microglia (ESC-MG) revealed a significant enrichment in the *Herpes simplex virus 1 infection* pathway.


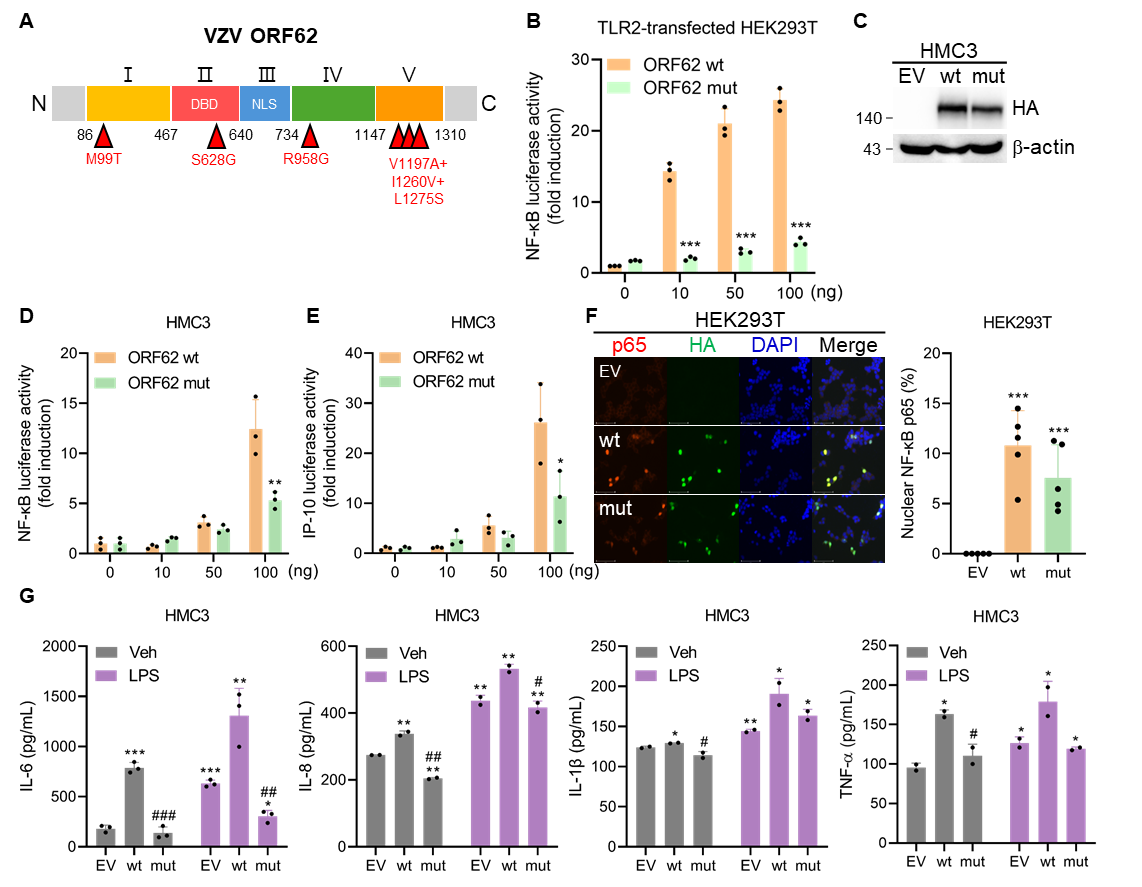


**Figure S7. VZV ORF62-induced NF-κB-mediated inflammatory response**

**A** Schematic representation of the mutation sites including M99T, S628G, R958G, V1197A, I1260V, L1275S in VZV ORF62 from MAV strains. **B** TLR2-expressing HEK293T cells were co-transfected with the indicated amounts of HA-tagged VZV ORF62 wild-type (wt) or mutant (mut) plasmids, along with NF-κB luciferase reporter plasmids for 24 h. After 24 h transfection, cells were treated with TNF-α for 6 h. Relative luciferase activity was measured. **C** Transfection efficiency of HA-tagged VZV ORF62 plasmids was confirmed by immunoblots. β-actin was used as a loading control. **D, E** HMC3 cells were transfected with varying concentrations of VZV ORF62 plasmids, along with NF-κB or IP-10 luciferase reporter plasmids for 24 h. Relative luciferase activity was measured. **F** HEK293T cells transfected with HA-tagged VZV ORF62 plasmids and treated with TNF-α. Cells were stained for NF-κB p65 (red) and HA (green). Nucleus was stained with DAPI (blue). Scale bar = 100 μm. The right graph represents the number of cells with nuclear NF-κB p65 positive staining. **G** The secretion levels of IL-6, IL-8, IL-1β, and TNF-α were measured after the treatment with vehicle (Veh) or LPS by ELISA. Statistical significance was determined as **p* < 0.05, ***p* < 0.01, ****p* < 0.001 compared to the EV-transfected or Veh-treated cells. ^#^*p* < 0.05, ^##^*p* < 0.01, ^###^*p* < 0.001 compared to the wt-transfected cells.


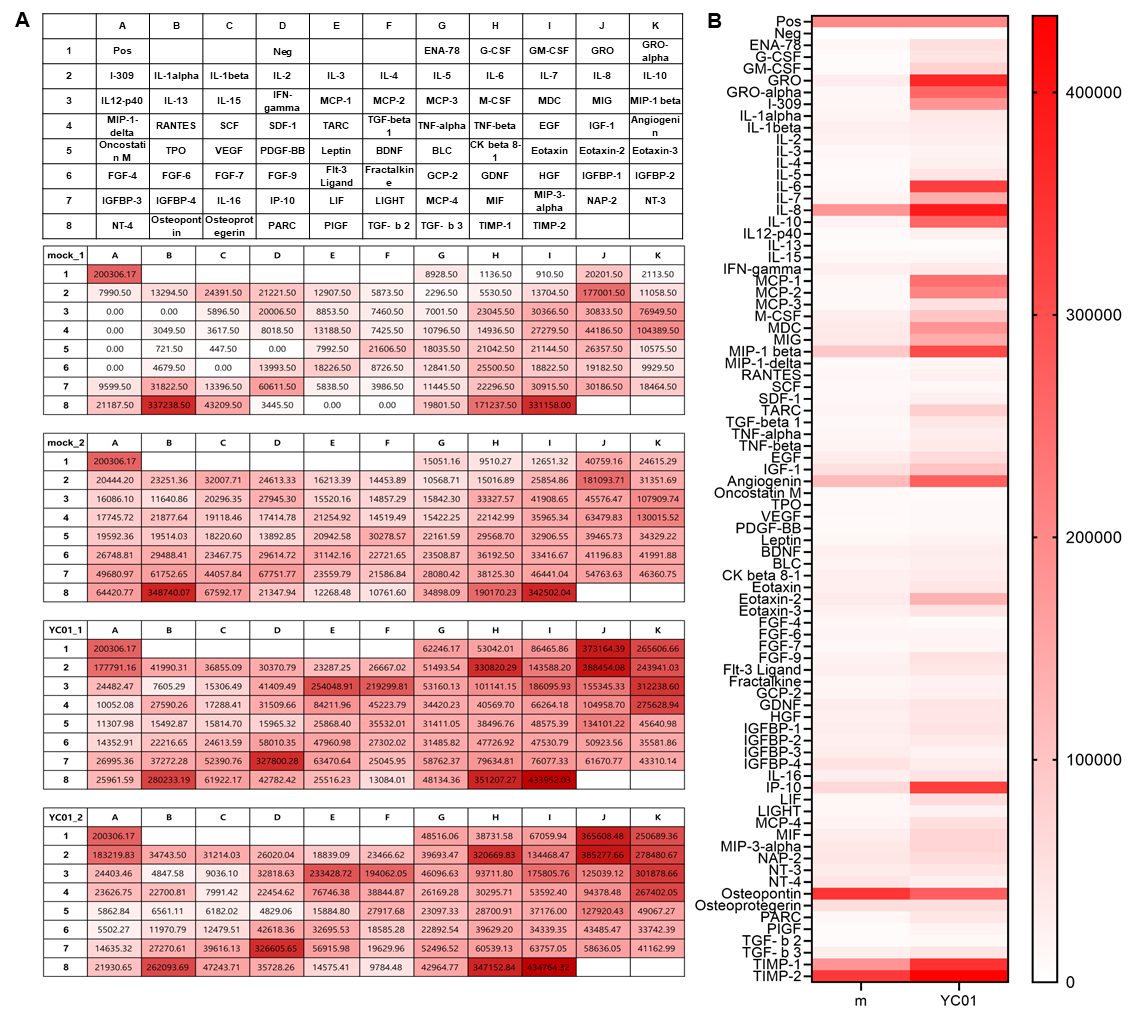


**Figure S8. Proinflammatory cytokine profiling of VZV-infected microglia by human cytokine antibody array**

**A** Representative cytokines detected by the human cytokine antibody array are shown. **B** A heatmap of the normalized array data for 80 cytokines is shown. POS denotes positive control and Neg denotes negative control.


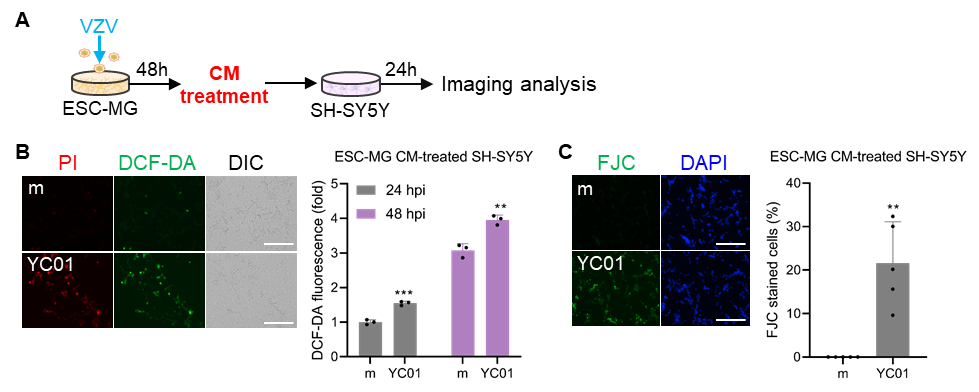


**Figure S9. Secretome from YC01-infected ESC-MG triggers ROS production and degenerating neurons in SH-SY5Y cells**

**A** A schematic diagram of the experimental workflow is shown. Conditioned media (CM) was collected from ESC-MG infected with mock (m) or VZV (YC01) at 48 hpi and subsequently applied to SH-SY5Y cells. **B** SH-SY5Y cells were stained with propidium iodide (PI) or 2’,7’-dichlorofluorescein diacetate (DCF-DA) to assess apoptotic cell death or reactive oxygen species (ROS) production. Scale bar = 200 μm. Quantification graphs are shown (mean ± SD, n=3). **C** SH-SY5Y cells were stained with Fluoro-Jade C (FJC) to visualize degenerating neurons. The percentage of FJC-positive cells was quantified (mean ± SD, n=3). Scale bar = 200 μm. **p < 0.01, ***p < 0.001 compared to m-infected group.
